# Pharmacokinetics and pharmacodynamics of pozelimab alone or in combination with cemdisiran in non-human primates

**DOI:** 10.1371/journal.pone.0269749

**Published:** 2022-06-16

**Authors:** Kishor Devalaraja-Narashimha, Cong Huang, Marc Cao, Ya Ping Chen, Anna Borodovsky, William C. Olson, Lori G. Morton, Marc W. Retter

**Affiliations:** 1 Regeneron Pharmaceuticals, Inc., Tarrytown, New York, United States of America; 2 Alnylam Pharmaceuticals, Inc., Cambridge, Massachusetts, United States of America; Laurentian University, CANADA

## Abstract

Paroxysmal nocturnal hemoglobinuria (PNH) is a rare disease caused by uncontrolled complement activation; effective and approved treatments include terminal complement inhibition. This study assessed whether combination cemdisiran (an investigational N-acetylgalactosamine-conjugated RNAi therapeutic that suppresses liver production of complement component C5) and pozelimab (an investigational fully human monoclonal antibody against C5) results in more effective and durable complement activity inhibition than the individual agents alone in non-human primates. Cynomolgus monkeys received a single subcutaneous injection of cemdisiran (5 or 25 mg/kg), pozelimab (5 or 10 mg/kg), or combination cemdisiran and pozelimab (5+5 mg/kg, 5+10 mg/kg, or 25+10 mg/kg, respectively). When given in combination, pozelimab was administered 2 weeks after cemdisiran dosing. Pharmacokinetics and *ex vivo* pharmacodynamic properties were assessed. The half-life of pozelimab alone was 12.9–13.3 days; this increased to 19.6–21.1 days for pozelimab administered in combination with cemdisiran. In *ex vivo* classical pathway hemolysis assays (CH_50_), pozelimab + cemdisiran combinations achieved durable and more complete suppression of complement activity (8–13 weeks) vs monotherapy of either agent. Cemdisiran monotherapy demonstrated dose-dependent suppression of total C5 concentrations, with the higher dose (25 mg/kg) achieving >90% maximum suppression. Total C5 concentrations after administration of pozelimab + cemdisiran combinations were similar compared with administration of cemdisiran alone. The combination of pozelimab + cemdisiran mediates complement activity inhibition more efficiently than either pozelimab or cemdisiran administered alone. The pharmacokinetic/pharmacodynamic profile of combination pozelimab + cemdisiran in non-human primates appears suitable for further clinical investigation as a potential long-acting treatment for PNH and other complement-mediated diseases.

## Introduction

Complement component C5 is a clinically validated target for several rare diseases, including paroxysmal nocturnal hemoglobinuria (PNH) [1], atypical hemolytic uremic syndrome [2], neuromyelitis optica [3], and a subset of patients with generalized myasthenia gravis [4]. PNH is a rare disease characterized by chronic intravascular hemolysis caused by uncontrolled complement activation [5]. This genetically acquired condition results from a somatic mutation in the phosphatidylinositol glycan class A (*PIGA*) gene during clonal expansion of hematopoietic stem cells, causing a reduction in glycosylphosphatidylinositol (GPI)-anchored complement regulatory proteins on the surface of red blood cells (RBCs) [5, 6]. Uncontrolled complement activation in PNH patients results in the primarily clinical manifestation of chronic hemolysis, as well as an increased risk of thromboembolism, leading to target organ damage and death [7, 8].

For patients with classic PNH, effective and approved treatments include terminal complement inhibition [9]. Two humanized monoclonal antibodies that target C5, eculizumab and ravulizumab, have been approved for the treatment of adults with PNH. Limitations of eculizumab treatment include the requirement for intravenous (IV) dosing every 2 weeks, which is burdensome to patients, and the fact that 25–35% of patients with PNH treated with eculizumab continue to require RBC transfusions due to breakthrough hemolysis, opsonization-mediated extravascular hemolysis, and bone marrow failure [10]. Furthermore, patients with PNH who received eculizumab required adjustments in dose and treatment intervals to maintain complete C5 blockade [9]. In addition, rare polymorphisms in the C5 protein (eg, R885H/C) result in a lack of efficacy for eculizumab [11].

Ravulizumab, approved by the US Food and Drug Administration in 2018, demonstrated non-inferiority to eculizumab in phase 3 studies of adults with PNH who were C5 inhibitor-naïve when administered IV every 8 weeks [12, 13]. However, hemolytic breakthroughs were still experienced in 4% and 10.7% of patients treated with ravulizumab and eculizumab, respectively [13], emphasizing the need to maintain essentially complete inhibition of C5 at all times. A limitation of ravulizumab treatment includes the need for IV dosing over several hours, which is burdensome to patients. In addition, ravulizumab was engineered from eculizumab and both bind to the same epitope, so ravulizumab is likely to have the same lack of efficacy to the C5 protein polymorphism (R885H/C) [14, 15]. Thus, there is an unmet medical need for treatments that prevent events of breakthrough hemolysis in patients with PNH and which offer a less burdensome treatment regimen.

Pozelimab is an investigational fully human monoclonal antibody shown to bind wild-type and variant (R885H/C) human C5 protein [16]; it demonstrated prolonged pharmacokinetic and pharmacodynamic profiles in non-human primates, and blocked complement-mediated hemolytic activity *ex vivo* for at least 35 days following a single 15 mg/kg subcutaneous (SC) dose [16]. In a phase 1 study in healthy volunteers (ClinicalTrials.gov identifier, NCT03115996), complement activation was robustly inhibited by frequent high doses of pozelimab (loading dose of pozelimab 15 mg/kg IV followed by four repeat doses of pozelimab 400 mg SC administered once weekly) [17]. Pozelimab is currently being evaluated for efficacy and safety in patients with PNH (ClinicalTrials.gov identifier, NCT03946748).

Cemdisiran, an investigational N-acetylgalactosamine-conjugated RNA interference (RNAi) therapeutic that reduces liver production of C5, demonstrated durable suppression of circulating levels of C5 following single and multiple SC doses in healthy volunteers (ClinicalTrials.gov identifier, NCT02352493) [18]. However, for patients with PNH who were eculizumab-naïve, levels of lactate dehydrogenase (an exploratory objective of the trial) remained above the treatment goal of <1.5 times the upper limit of normal, suggesting that cemdisiran monotherapy may not be sufficient to provide clinical benefit in PNH [18]. Because of the longer time to effect and incomplete C5 suppression with RNAi therapeutics, as well as high and frequent dosing due to target load with anti-C5 antibodies, combining the complementary approaches of C5 reduction and antibody-mediated inhibition may be a better clinical strategy to offer a more patient-friendly infrequent SC dosing regimen while potentially minimizing intravascular hemolysis breakthroughs.

The objective of the current study was to assess whether reducing circulatory C5 levels with cemdisiran lowers the threshold of C5 blockade required by pozelimab and allows for a more robust and durable inhibition of C5 activity when combined than the inhibition observed with the individual agents alone. Accordingly, a single SC dose of multiple combinations of pozelimab + cemdisiran was compared with administration of pozelimab or cemdisiran alone in non-human primates with reference to the pharmacokinetics and *ex vivo* pharmacodynamic properties.

## Methods

### Non-human primate studies

Cynomolgus monkeys were provided by and housed at AltaSciences Preclinical Seattle LLC. AltaSciences Preclinical Seattle LLC laboratories are fully accredited by the Association for Assessment and Accreditation of Laboratory Animal Care (AALAC), in compliance with the Animal Welfare Act (Code of Regulations, Title 9), the Public Health Service Policy on Humane Care and Use of Laboratory Animals from the Office of Laboratory Animal Welfare, and the Guide for the Care and Use of Laboratory Animals from the National Research Council under Regeneron study number R3918-PK-18105. The study was conducted according to a protocol, and procedures were approved by an Institutional Animal Care and Use Committee. Veterinary care was available throughout the study by an attending laboratory animal veterinarian. In accordance with applicable animal welfare guidelines, any medical treatment necessary to prevent unacceptable pain and suffering, including euthanasia, was the sole responsibility of the attending veterinarian. This was a single-dose pharmacokinetic study, with animals dosed SC and bled at various timepoints as noted below. No other procedures were performed on the animals.

Animals (aged 2–4 years, ranging 2–4.9 kg in weight) were housed at AltaSciences, a fully AAALAC-accredited facility, in stainless steel cages, individually housed and co-mingled (same dosing group together) according to laboratory standard operating procedures (SOPs). Animal rooms were temperature-controlled with 12 hours light/12 hours dark cycles. Animals were provided feed and water (*ad libitum*), and other appropriate treats. Animals were provided PMI LabDiet^®^ Fiber-Plus^®^ Monkey Diet 5049 biscuits twice daily. Food evaluation was performed once daily in the morning prior to feeding, beginning on Week –1. A visual inspection for overall appetite was performed; the documentation consisted of a positive entry of the clinical sign “inappetent” if the animal showed no interest in its food ration. Fruits, vegetables, and other dietary supplements, as well as in-cage enrichment devices (such as perches, floor toys, foraging devices, and/or hanging devices) were provided throughout the course of the study in accordance with applicable laboratory SOPs. Animals were monitored twice daily via cage-side observations for general health and wellness. Cage-side clinical observations were recorded once daily, beginning on the second day of acclimation for each animal in the morning prior to room cleaning. Additional clinical observations were performed as necessary to properly monitor the animals’ health condition. Upon completion of the in-life portion of the study, all animals were returned to the laboratory stock colony (no animals were euthanized).

### Pharmacokinetic/pharmacodynamic study design

Each of 29 male cynomolgus monkeys were assigned to one of seven groups: Groups 1–2, n = 3 animals/group; Groups 3–4, n = 4 animals/group; and Groups 5–7, n = 5 animals/group (Table 1). Pre-study concentrations of C5 in monkey serum were determined using a commercially available enzyme-linked immunosorbent assay (ELISA) kit including reference standards (complement C5 human ELISA kit ab125963; Abcam, Cambridge, MA). Monkeys selected for inclusion in the study had an average C5 concentration of approximately 32 μg/mL (as determined using the standard curve from the specific lot of the ELISA kit).

**Table 1:** Pharmacokinetic/pharmacodynamic study design of pozelimab and cemdisiran combination in male cynomolgus monkeys.

Group	Animals, n	Agent*	Dose day	Dose route	Dose level (mg/kg)	Dose concentration (mg/mL)
1	3	Cemdisiran	1	SC	5	5
2	3	Cemdisiran	1		25	25
3	4	Pozelimab	1		5	5
4	4	Pozelimab	1		10	10
5	5	Cemdisiran + pozelimab	1 15		5 5	5 5
6	5	Cemdisiran + pozelimab	1 15		5 10	5 10
7	5	Cemdisiran + pozelimab	1 15		25 10	25 10

SC, subcutaneous.

*Administered as a single SC injection.

Cynomolgus monkeys received a single SC injection of cemdisiran alone (5 or 25 mg/kg), pozelimab alone (5 or 10 mg/kg), or cemdisiran + pozelimab combination (5 + 5 mg/kg, 5 + 10 mg/kg, or 25 + 10 mg/kg). The pozelimab in combination was administered 2 weeks after cemdisiran. The target dose volume for each group was 1 mL/kg.

Blood samples to measure serum concentrations of total pozelimab, C5, and *ex vivo* blockade of RBC hemolysis were collected at baseline (pre-dose) and 0.5 (Groups 3–7 only), 4, 8, 24, 48, 72, 168, 240, 336, 432, 504, 672, 840, 1008, 1176, 1344, 1512, and 1680 hours (Day 70) post-dose, or 1848, 2016, 2184, and 2352 hours (Day 98) post-dose (Groups 5–7 only).

### Assessments

#### Serum concentration of pozelimab

Concentrations of total pozelimab in cynomolgus monkey serum were determined using a validated ELISA assay [16]. The lower limit of quantitation of the assay was 0.078 μg/mL in neat monkey serum.

#### Classical pathway hemolysis assay

*Ex vivo* classical pathway (CP) hemolytic activity (CH50) in serum as a function of time was determined in a cell-based assay using hemolysin-sensitized sheep erythrocytes [19]. Hemolysis assays were performed as previously reported [20], with slight modifications (S1 File). The CP was activated when serum was incubated with sheep erythrocytes pre-coated with rabbit anti-sheep erythrocyte antibodies (hemolysin); hemolysis of sheep erythrocytes was quantified by measuring the optical density at 412 nm, which is proportional to the amount of hemoglobin released into the supernatant.

#### Serum concentration of total cynomolgus monkey C5 using liquid chromatography-multiple reaction monitoring-mass spectrometry

Concentrations of total cynomolgus monkey C5 in serum were quantified using liquid chromatography-multiple reaction monitoring-mass spectrometry (LC-MRM-MS) which has been described previously [16]. Assay details are described in the S1 File. The lower limit of quantification of the assay was 0.977 μg/mL.

### Data analyses

Total pozelimab concentrations were analyzed by descriptive statistics and summarized by time and treatment group, with all concentrations rounded to three significant figures. Pharmacokinetic variables were analyzed by noncompartmental analysis (NCA) (Phoenix^®^, WinNonlin^®^ [Version 6.4, Certara L.P., Princeton, NJ]) using an extravascular model. Linear trapezoidal rule and linear interpolation with uniform weighting were used in the model settings. Values below the limit of quantitation were considered as zero during the NCA. Changes in hemolytic activity following cemdisiran and/or pozelimab dosing, compared with the respective pre-dose baseline levels, were assessed by analysis of variance followed by Dunnett’s multiple comparisons post hoc test. Differences were considered to be significant when the *p* value was ≤0.05.

## Results

### Non-human primate pharmacokinetics of pozelimab alone or in combination with cemdisiran

**Fig 1:**
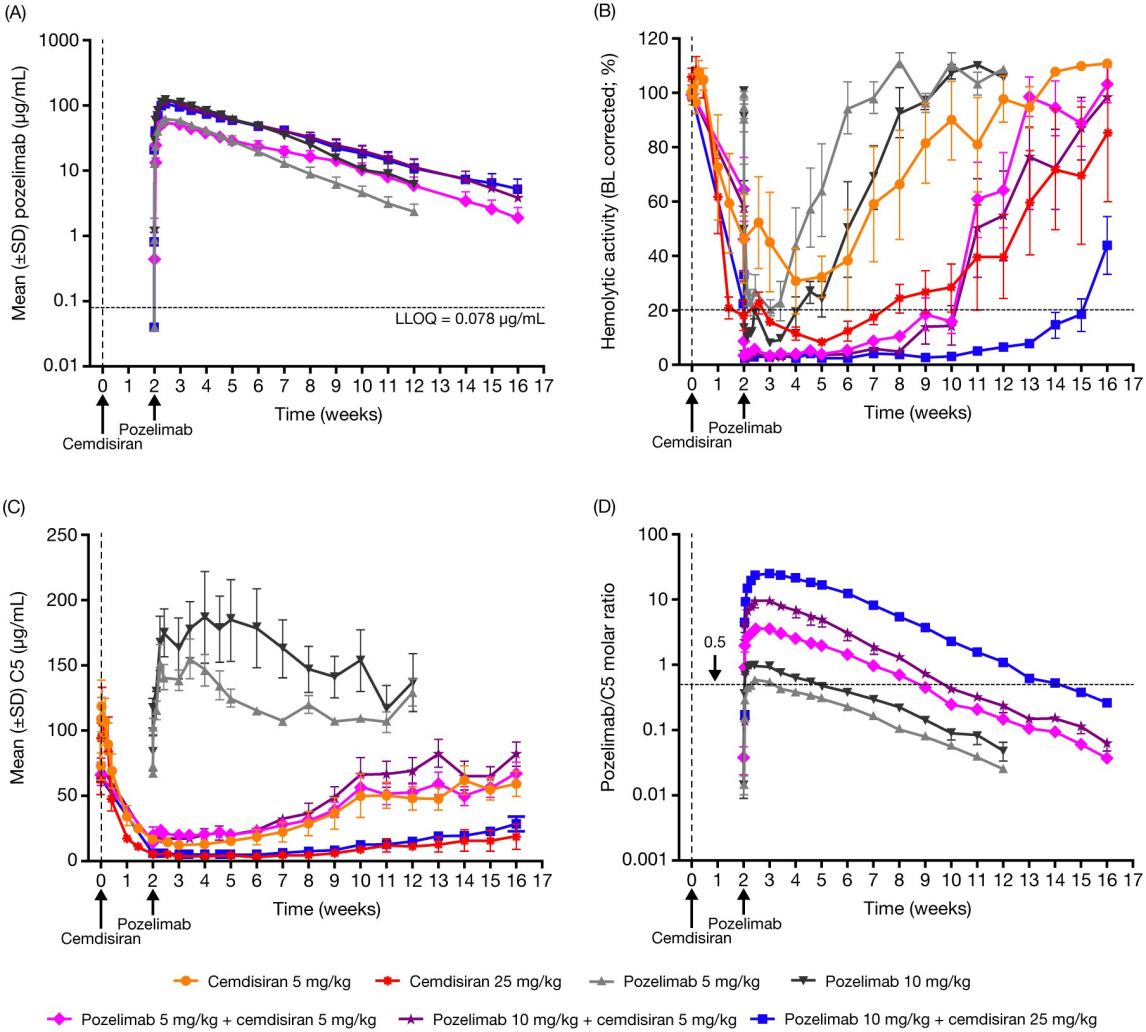
(A) Mean total concentrations of pozelimab in serum after single subcutaneous injection of pozelimab alone or in combination with cemdisiran in male cynomolgus monkeys;* (B) [baseline corrected] *ex vivo* classic pathway hemolysis in serum from cynomolgus monkeys administered pozelimab alone or in combination with cemdisiran; (C) total mean C5 concentrations in serum from cynomolgus monkeys administered pozelimab alone or in combination with cemdisiran; and (D) pozelimab/C5 molar ratios in serum over time. BL, baseline; C5, complement component 5; LLOQ, lower limit of quantitation; SD, standard deviation. *Total pozelimab concentrations below the LLOQ (<0.078 μg/mL) were imputed as LLOQ/2 (0.039 μg/mL), and concentration values considered by the investigator to be outliers were excluded for all animals from Groups 5–7 at Day 78. Concentration values considered by the study investigator to be impacted by anti-drug antibodies were excluded; excluded values were from one animal in the pozelimab 10 mg/kg group.

Following SC administration of pozelimab alone or in combination with cemdisiran, concentration–time profiles of total pozelimab were characterized by an absorption phase followed by a single elimination phase over the study period (Fig 1A; Table 2; raw data provided in S1 Table in S1 File). For pozelimab alone, dose-proportional increases in peak concentration (C_max_) were observed. When C_max_ was normalized to dose, values were within 1.0-fold and 1.1-fold for pozelimab alone or in combination with cemdisiran, respectively. Similarly, dose proportional increases at all dose levels were observed for the area under the concentration–time curve (AUC) extrapolated to infinity. However, there was a visually apparent slower elimination of pozelimab in the combination dose groups compared to the pozelimab-alone groups that can be observed in the divergence of the concentration–time profiles starting at approximately Week 5 for the 5 mg/kg pozelimab alone group compared with the combination group profiles, and approximately Week 7 for the 10 mg/kg pozelimab alone group compared with the combination group profiles.

**Table 2:** Mean pharmacokinetic parameters of total pozelimab in serum following a single subcutaneous injection of pozelimab alone or in combination with cemdisiran in male cynomolgus monkeys*.

Parameter, mean (SD)	Pozelimab 5 mg/kg (n = 4)	Pozelimab 10 mg/kg (n = 4)	Pozelimab 5 mg/kg + cemdisiran 5 mg/kg (n = 5)	Pozelimab 10 mg/kg + cemdisiran 5 mg/kg (n = 5)	Pozelimab 10 mg/kg + cemdisiran 25 mg/kg (n = 5)
C_max_, μg/mL	63.0 (4.8)	123 (9.3)	56.1 (3.2)	114 (9.5)	107 (10.0)
C_max_/dose, (μg/mL)/(mg/kg)	12.6 (1.0)	12.3 (0.9)	11.2 (0.6)	11.4 (1.0)	10.7 (1.0)
T_max_, days	3.0 (0)	3.0 (0)	3.6 (2.0)	3.60 (2.0)	2.80 (0.4)
AUC_inf_, days*(μg/mL)	1510 (195)	3310 (528)	1810 (249)	3650 (455)	3570 (551)
AUC_inf_/dose, days*(μg/mL)/(mg/kg)	303 (39)	331 (53)	361 (50)	365 (46)	357 (55)
T_1/2_, days	13.3 (1.1)	12.9 (4.8)	19.9 (2.3)	19.6 (2.1)	21.1 (3.2)

AUC, area under concentration-time curve; AUC_inf_, AUC from time zero extrapolated to infinity; C_max_, peak concentration; d, day; n, number of animals per group; SD, standard deviation; T_1/2_, elimination half-life; T_max_, time to C_max_.

*Concentration values considered by the investigator to be outliers were excluded for all animals from Groups 5–7 at Day 78. Concentration values considered by the study investigator to be impacted by anti-drug antibodies were excluded (n = 1 in the pozelimab 10 mg/kg group).

Mean half-life ranged from 12.9 to 13.3 days for pozelimab administered alone and increased to 19.6 to 21.1 days for pozelimab administered in combination with cemdisiran, indicating that cemdisiran-mediated reduction in C5 concentrations in serum in the combination groups leads to slower elimination of pozelimab and a longer relative pozelimab half-life, likely due to a reduction in the degree of target-mediated clearance of pozelimab.

### Non-human primate pharmacodynamics of pozelimab alone or in combination with cemdisiran

The capacity of pozelimab alone, cemdisiran alone, or combination pozelimab and cemdisiran to block C5-dependent CP hemolytic activity in serum over time was assessed by *ex vivo* CH_50_ assay (Fig 1B; raw data provided in S2 Table in S1 File). Administration of pozelimab or cemdisiran alone at 5 mg/kg was not able to achieve the target suppression of hemolysis of >80%, commonly considered to represent effective complement blockade *in vivo* [21]. At the higher dose, pozelimab 10 mg/kg achieved target suppression of hemolysis for 2 weeks. Administration of cemdisiran 25 mg/kg required 2 weeks to achieve target suppression that was maintained for 5 weeks.

For all combination dose groups, target suppression of hemolysis (>80%) was achieved within 4 hours of pozelimab administration. Suppression of hemolysis was maintained for 8 weeks with cemdisiran + pozelimab combinations of 5 + 5 mg/kg and 5 + 10 mg/kg. At the highest combination dose, cemdisiran + pozelimab 25 + 10 mg/kg, suppression of hemolysis was extended to 13 weeks.

Pharmacodynamics were also assessed by analyzing total C5 concentrations using LC-MRM-MS. Following administration of pozelimab alone, mean concentrations of C5 increased to a maximum of 2.2-fold over baseline at 1–2 weeks post-dose and then decreased over 10 weeks. Higher mean concentrations of C5 were observed post-treatment with pozelimab at 10 mg/kg compared with pozelimab 5 mg/kg (Fig 1C; raw data provided in S3 Table in S1 File). Based on group mean values, >80% inhibition of CP hemolysis generally corresponded to a pozelimab/C5 molar ratio >0.6 (Fig 1D; raw data provided in S4 Table in S1 File).

Following administration of cemdisiran alone, at 4–8 hours post-dose the total mean C5 concentrations were increased by 1.6–1.7-fold before returning to baseline levels by Day 3 (Fig 1C). Then, over an extended period, the concentrations of total C5 were reduced from baseline (~70 μg/mL; LC-MRM-MS assay) to <25 μg/mL and <5 μg/mL with cemdisiran 5 mg/kg and cemdisiran 25 mg/kg alone or in combination, respectively. C5 concentrations recovered to baseline at approximately Week 10 in the cemdisiran 5 mg/kg groups, in contrast to the cemdisiran 25 mg/kg groups where C5 concentrations recovered to only 30–45% of baseline by Week 16 (alone or in combination, respectively).

## Discussion

Inhibition of C5 is a clinically validated mechanism to treat PNH, which is characterized by uncontrolled terminal complement activation. Preliminary clinical data demonstrated that the combination of an anti-C5 antibody and cemdisiran has the potential to address some of the limitations (eg, dosing, breakthrough hemolysis) with currently approved anti-C5 antibody treatments for patients with PNH [18]. In this study, dose-proportional increases in pozelimab exposure (C_max_ and AUC) in non-human primates were observed across all dose levels when administered alone or in combination with cemdisiran. The half-life (12.9–13.3 days) of pozelimab in this study was consistent with published values in monkeys (~14 days) [16]. The longer half-life of pozelimab when administered in combination with cemdisiran compared with pozelimab alone is consistent with reduced C5-mediated clearance of pozelimab upon the silencing of liver C5 expression by cemdisiran. The results of this proof-of-concept study demonstrate the merits of combining antibody and gene silencing therapies, and furthermore show that this approach has the potential to offer long-acting, self-administrable therapy for PNH and other complement-mediated diseases.

In the *ex vivo* CP hemolysis assay, the pozelimab + cemdisiran combinations achieved durable and more complete suppression of complement activity (8–13 weeks) compared with monotherapy of either agent (maximum 5 weeks). Specifically, the pharmacodynamic effect of combination pozelimab 5 mg/kg + cemdisiran 5 mg/kg was more profound with more durable hemolytic activity suppression than the sub-optimal response observed with either monotherapy at 5 mg/kg. In addition, complement inhibition by the combination of pozelimab 10 mg/kg with cemdisiran 5 or 25 mg/kg was extended by 6 and 11 weeks, respectively, compared with the pozelimab 10 mg/kg monotherapy group.

Both doses of cemdisiran alone demonstrated dose-dependent suppression of total C5 concentrations, with the higher dose (25 mg/kg) achieving maximum suppression of >90%, consistent with previously published results [22]. Similar to published reports [16], total C5 concentrations increased 2.2-fold and then slowly declined after administration of pozelimab alone. The concentration of total C5 after administration of pozelimab + cemdisiran combinations was similar compared with administration of cemdisiran alone. The function of C5 is expected to be blocked entirely at a monoclonal antibody:C5 ratio of ≥0.5 since each monoclonal antibody molecule binds to two C5 molecules. The current study demonstrated that a monoclonal antibody:C5 ratio of ≥0.6 was associated with >80% inhibition of CP hemolysis. The ratio was maintained at this level for an extended duration in the combination groups, likely due to a cemdisiran-mediated reduction in C5 concentrations.

Collectively, these data indicate that, through complementary reduction of circulating C5 concentrations in serum by cemdisiran, pozelimab completely inhibits the activity of the remaining free C5. The combination of pozelimab and cemdisiran offered more effective and long-lasting inhibition of C5 activity than either pozelimab or cemdisiran administered alone. Overall, the pharmacokinetic/pharmacodynamic profile of the pozelimab + cemdisiran combination in non-human primates appears suitable for further investigation as a potential treatment for PNH and other complement-mediated diseases. In addition, combining antibody and gene-silencing therapies has the potential to optimally address numerous grievous diseases characterized by high target burden and the need for complete target inhibition.

## Supporting information

S1 File (DOCX)
