# Supplementary material for: Pharmacokinetics and pharmacodynamics of pozelimab alone or in combination with cemdisiran in non-human primates
Source: PLoS One. 2022 Jun 16;17(6):e0269749. doi: 10.1371/journal.pone.0269749 (PMC9202903; doi:10.1371/journal.pone.0269749)
Supplement: S1 File — (DOCX) [file pone.0269749.s001.docx]

# Supplementary materials

## Classical pathway hemolysis assay

*Ex vivo* classic pathway hemolytic activity (CH_50_) in serum as a function of time was determined in a cell-based assay using hemolysin-sensitized sheep erythrocytes (SRBCs) [1]. SRBCs were washed and resuspended at 1 × 10^9^ cells/mL in gelatin veronal buffer with CaCl_2_ and MgCl_2_ (GVB++). Then, SRBCs were sensitized by incubation with 1.5 mg/mL hemolysin at 37⁰C for 20 minutes. Aliquots (100 μL each) of sensitized SRBCs (2 × 10^8^ cells) and test serum (final 5%) were added to a V-bottom 96-well plate. Sensitized SRBCs in GVB++ determined background hemolysis (no test serum). Cells were lysed by the addition of distilled water at 37°C to determine the maximal hemolysis signal. To quantify hemolysis, cells were pelleted by centrifugation (1250 × g) at 4°C for 7 minutes. Then, 100 μL of the supernatant from each well was transferred to a clean 96-well plate, and OD_412_ was determined using a Spectramax microplate reader (Molecular Devices, LLC, San Jose, CA). Hemolytic activity was calculated as follows:

$$\% hemolysis= \frac{Test sample hemolysis \left( OD412 \right)-background hemolysis (OD412)}{Maximal hemolysis \left( OD412 \right)-background hemolysis (OD412)}$$

$$\% inhibition of hemolysis=100- \frac{\% hemolysis (at time X postdose)}{\% hemolysis (predose)} \times100$$

## Serum concentration of total cynomolgus monkey C5 using liquid chromatography-multiple reaction monitoring-mass spectrometry

Concentrations of total cynomolgus monkey C5 in serum were quantified using liquid chromatography-multiple reaction monitoring-mass spectrometry (LC-MRM-MS) which has been described previously [2]. Concentrations of total cynomolgus monkey C5 in serum were determined by measuring the concentration of a 10-amino acid peptide present in the C5 protein (AA 1129-1138: LQGTLPVEAR). Theoretically, this method could also detect the cleaved product of C5, C5b; however, concentrations of C5b would be negligible due to the instability of free C5b in serum and with the majority of C5b bound as membrane attack complexes on cell surfaces [3, 4]. Briefly, the surrogate human C5 peptide (AA 1129–1138) labeled at the C-terminus arginine residue (LQGTLPVEAR-^13^C_6_^15^N_4_) was used as an internal standard. The reference standard (unlabeled human C5 peptide) was serially diluted (1:2) from a starting concentration of 500 μg/mL in mouse serum and used for calibration. Mouse serum was also used as a negative control (blank). Calibration standards, internal standard, blanks (mouse serum), and serum samples (10 μL) were denatured in 80 μL of 8 M urea/20 mM Tris(2-carboxyethyl)phosphine buffer at 37⁰C for 1 hour. Then, 10 μL of internal standard (25 ng/μL) in 100 mM iodoacetamide was added to all samples for alkylation at room temperature for 30 minutes in the dark. Finally, all samples were diluted to a final volume of 500 μL with 50 mM ammonium bicarbonate before digestion with trypsin (1:20 w/w) overnight at 37°C. The tryptic peptide (LQGTLPVEAR) derived from C5 was detected and quantified by LC-MRM-MS using a Waters Xevo TQ-XS with ACQUITY UPLC system (Waters, Milford, MA). Each processed sample (10 μL) was injected on a pre-equilibrated ACQUITY UPLC BEH peptide C18 column, with a flow rate of 0.4 mL/min (mobile phase A: 0.1% formic acid in water; mobile phase B: 0.1% formic acid in acetonitrile). Retention time and peak area were determined using Masslynx Analyst Data software (Waters, Milford, MA). Concentrations of C5 were calculated from the plotted calibration curve (constructed by plotting the peak area ratio of C5 reference standard to internal standard vs the nominal concentration of spiked C5 reference standard) fitted using linear regression with 1/x weighting factor. The lower limit of quantification of the assay was 0.977 μg/mL.

# References

1. Costabile M. Measuring the 50% haemolytic complement (CH50) activity of serum. J Vis Exp 2010:1923. doi: 10.3791/1923. PMID: 20351687; PubMed Central PMCID: PMCPMC3168207.

2. Latuszek A, Liu Y, Olsen O, Foster R, Cao M, Lovric I, et al. Inhibition of complement pathway activation with Pozelimab, a fully human antibody to complement component C5. PLoS One 2020;15:e0231892. doi: 10.1371/journal.pone.0231892.

3. Kolb WP, Müller-Eberhard HJ. The membrane attack mechanism of complement. Verification of a stable C5-9 complex in free solution. J Exp Med 1973;138:438-51. doi: 10.1084/jem.138.2.438. PMID: 4719679; PubMed Central PMCID: PMCPMC2139397.

4. Rawal N, Pangburn MK. C5 convertase of the alternative pathway of complement: Kinetic analysis of the free and surface-bound forms of the enzyme*. Journal of Biological Chemistry 1998;273:16828-35. doi: 10.1074/jbc.273.27.16828.

Supplementary Table 1. Mean concentration of total pozelimab in serum following a single SC injection of pozelimab alone or pozelimab in combination with cemdisiran in the male cynomolgus monkey

| **Total Pozelimab Concentrations** | | | | | | | | | | | | | | | | |  |
| --- | --- | --- | --- | --- | --- | --- | --- | --- | --- | --- | --- | --- | --- | --- | --- | --- | --- |
| **Pozelimab 5 mg/kg SC** | | | | | | | | | | | | | | | | |  |
| **Study Day** | | **Time Post Dose (h)** | **n** | | | | | | | **Mean** | **SD** | | **CV** | | | |  |
|  |  |  |  |  |  |  |  |  |  | **µg/mL** | | | **%** | | | |  |
| D1 | | 0 | 4 | | | | | | | BLQ | NC | | NC | | | |  |
|  |  | 0.5 | 4 | | | | | | | 0.872 | 0.558 | | 64.0 | | | |  |
|  |  | 4 | 4 | | | | | | | 13.1 | 3.46 | | 26.5 | | | |  |
|  |  | 8 | 4 | | | | | | | 23.8 | 2.49 | | 10.4 | | | |  |
| D2 | | 24 | 4 | | | | | | | 40.8 | 3.50 | | 8.57 | | | |  |
| D3 | | 48 | 4 | | | | | | | 57.3 | 2.13 | | 3.72 | | | |  |
| D4 | | 72 | 4 | | | | | | | 63.0 | 4.80 | | 7.63 | | | |  |
| D8 | | 168 | 4 | | | | | | | 58.5 | 6.46 | | 11.1 | | | |  |
| D11 | | 240 | 4 | | | | | | | 49.9 | 6.73 | | 13.5 | | | |  |
| D15 | | 336 | 4 | | | | | | | 42.0 | 5.73 | | 13.6 | | | |  |
| D19 | | 432 | 4 | | | | | | | 33.8 | 3.93 | | 11.6 | | | |  |
| D22 | | 504 | 4 | | | | | | | 28.5 | 5.50 | | 19.3 | | | |  |
| D29 | | 672 | 4 | | | | | | | 19.8 | 4.22 | | 21.3 | | | |  |
| D36 | | 840 | 4 | | | | | | | 13.1 | 2.83 | | 21.6 | | | |  |
| D43 | | 1008 | 4 | | | | | | | 8.94 | 2.31 | | 25.8 | | | |  |
| D50 | | 1176 | 4 | | | | | | | 6.35 | 1.58 | | 24.8 | | | |  |
| D57 | | 1344 | 4 | | | | | | | 4.62 | 1.03 | | 22.3 | | | |  |
| D64 | | 1512 | 4 | | | | | | | 3.15 | 0.714 | | 22.7 | | | |  |
| D71 | | 1680 | 4 | | | | | | | 2.36 | 0.676 | | 28.6 | | | |  |
| **Total Pozelimab Concentrations** | | | | | | | | | | | | | | | | |  |
| **Pozelimab 10 mg/kg SC** | | | | | | | | | | | | | | | | |  |
| **Study Day** | | **Time Post Dose (h)** | **n** | | | | | | | **Mean** | **SD** | | **CV** | | | |  |
|  |  |  |  |  |  |  |  |  |  | **µg/mL** | | | **%** | | | |  |
| D1 | | 0 | 4 | | | | | | | BLQ | NC | | NC | | | |  |
|  |  | 0.5 | 4 | | | | | | | 1.15 | 0.681 | | 59.3 | | | |  |
|  |  | 4 | 4 | | | | | | | 29.5 | 6.43 | | 21.8 | | | |  |
|  |  | 8 | 4 | | | | | | | 57.6 | 11.3 | | 19.6 | | | |  |
| D2 | | 24 | 4 | | | | | | | 85.4 | 6.97 | | 8.17 | | | |  |
| D3 | | 48 | 4 | | | | | | | 114 | 8.62 | | 7.58 | | | |  |
| D4 | | 72 | 4 | | | | | | | 123 | 9.32 | | 7.60 | | | |  |
| D8 | | 168 | 4 | | | | | | | 113 | 12.8 | | 11.4 | | | |  |
| D11 | | 240 | 4 | | | | | | | 100 | 12.7 | | 12.7 | | | |  |
| D15 | | 336 | 4 | | | | | | | 85.3 | 8.19 | | 9.60 | | | |  |
| D19 | | 432 | 4 | | | | | | | 73.3 | 8.37 | | 11.4 | | | |  |
| D22 | | 504 | 4 | | | | | | | 62.2 | 9.73 | | 15.6 | | | |  |
| D29 | | 672 | 4 | | | | | | | 49.9 | 7.26 | | 14.6 | | | |  |
| D36 | 840 | | | 4 | | | | | 35.9 | | | 7.15 | | | | 19.9 | |
| D43 | 1008 | | | 4 | | | | | 25.0 | | | 6.65 | | | | 26.6 | |
| D50 | 1176 | | | 4 | | | | | 15.9 | | | 7.45 | | | | 47.0 | |
| D57 | 1344 | | | 4 | | | | | 10.4 | | | 5.90 | | | | 56.6 | |
| D64 | 1512 | | | 3 | | | | | 8.98 | | | 4.19 | | | | 46.7 | |
| D71 | 1680 | | | 3 | | | | | 6.19 | | | 2.99 | | | | 48.2 | |
| **Total Pozelimab Concentrations** | | | | | | | | | | | | | | | | | |
| **Pozelimab 5 mg/kg + Cemdisiran 5 mg/kg SC** | | | | | | | | | | | | | | | | | |
| **Study Day** | **Time Post Dose (h)** | | | **n** | | | | | **Mean** | | | **SD** | | | | **CV** | |
|  |  |  |  |  |  |  |  |  | **µg/mL** | | | | | | | **%** | |
| D1 | 0 | | | 5 | | | | | BLQ | | | NC | | | | NC | |
|  | 0.5 | | | 5 | | | | | 0.436 | | | 0.262 | | | | 60.1 | |
|  | 4 | | | 5 | | | | | 12.6 | | | 3.51 | | | | 27.9 | |
|  | 8 | | | 5 | | | | | 25.2 | | | 6.01 | | | | 23.8 | |
| D2 | 24 | | | 5 | | | | | 37.9 | | | 7.19 | | | | 19.0 | |
| D3 | 48 | | | 5 | | | | | 51.8 | | | 6.09 | | | | 11.7 | |
| D4 | 72 | | | 5 | | | | | 55.8 | | | 2.94 | | | | 5.27 | |
| D8 | 168 | | | 5 | | | | | 51.1 | | | 5.34 | | | | 10.5 | |
| D11 | 240 | | | 5 | | | | | 44.1 | | | 4.25 | | | | 9.63 | |
| D15 | 336 | | | 5 | | | | | 38.3 | | | 3.77 | | | | 9.85 | |
| D19 | 432 | | | 5 | | | | | 33.0 | | | 4.11 | | | | 12.5 | |
| D22 | 504 | | | 5 | | | | | 29.0 | | | 3.74 | | | | 12.9 | |
| D29 | 672 | | | 5 | | | | | 23.9 | | | 4.09 | | | | 17.1 | |
| D36 | 840 | | | 5 | | | | | 20.1 | | | 3.63 | | | | 18.0 | |
| D43 | 1008 | | | 5 | | | | | 16.3 | | | 3.64 | | | | 22.4 | |
| D50 | 1176 | | | 5 | | | | | 13.1 | | | 3.27 | | | | 24.9 | |
| D57 | 1344 | | | 5 | | | | | 10.4 | | | 2.76 | | | | 26.6 | |
| D64 | 1512 | | | 5 | | | | | 8.03 | | | 2.51 | | | | 31.2 | |
| D71 | 1680 | | | 5 | | | | | 5.97 | | | 1.95 | | | | 32.6 | |
| D78 | 1848 | | | 0 | | | | | NC | | | NC | | | | NC | |
| D85 | 2016 | | | 5 | | | | | 3.52 | | | 1.12 | | | | 31.8 | |
| D92 | 2184 | | | 5 | | | | | 2.65 | | | 0.848 | | | | 32.0 | |
| D99 | 2352 | | | 5 | | | | | 1.92 | | | 0.738 | | | | 38.4 | |
| **Total Pozelimab Concentrations** | | | | | | | | | | | | | | | | | |
| **Pozelimab 10 mg/kg + Cemdisiran 5 mg/kg SC** | | | | | | | | | | | | | | | | | |
| **Study Day** | **Time Post Dose (h)** | | | | **n** | | | **Mean** | | | **SD** | | | | **CV** | | |
|  |  |  |  |  |  |  |  | **µg/mL** | | | | | | | **%** | | |
| D1 | 0 | | | | 5 | | | BLQ | | | NC | | | | NC | | |
|  | 0.5 | | | | 5 | | | 1.46 | | | 0.568 | | | | 39.0 | | |
|  | 4 | | | | 5 | | | 26.7 | | | 11.5 | | | | 43.2 | | |
|  | 8 | | | | 5 | | | 45.4 | | | 19.8 | | | | 43.7 | | |
| D2 | 24 | | | | 5 | | | 76.9 | | | 17.6 | | | | 22.9 | | |
| D3 | 48 | | | | 5 | | | 106 | | | 18.9 | | | | 17.9 | | |
| D4 | 72 | | | | 5 | | | 112 | | | 12.0 | | | | 10.7 | | |
| D8 | 168 | | | | 5 | | | 102 | | | 5.20 | | | | 5.09 | | |
| D11 | 240 | | | | 5 | | | 91.8 | | | 6.01 | | | | 6.54 | | |
| D15 | 336 | | | | 5 | | | 79.5 | | | 8.84 | | | | 11.1 | | |
| D19 | 432 | | | | 5 | | | 68.3 | | | 11.0 | | | | 16.2 | | |
| D22 | 504 | | | | 5 | | | 61.4 | | | 8.91 | | | | 14.5 | | |
| D29 | 672 | | | | 5 | | | 48.6 | | | 6.76 | | | | 13.9 | | |
| D36 | 840 | | | | 5 | | | 40.8 | | | 4.90 | | | | 12.0 | | |
| D43 | 1008 | | | | 5 | | | 32.3 | | | 6.17 | | | | 19.1 | | |
| D50 | 1176 | | | | 5 | | | 24.9 | | | 5.69 | | | | 22.8 | | |
| D57 | 1344 | | | | 5 | | | 20.2 | | | 4.59 | | | | 22.7 | | |
| D64 | 1512 | | | | 5 | | | 15.4 | | | 3.52 | | | | 22.8 | | |
| D71 | 1680 | | | | 5 | | | 11.4 | | | 3.18 | | | | 27.8 | | |
| D78 | 1848 | | | | 0 | | | NC | | | NC | | | | NC | | |
| D85 | 2016 | | | | 5 | | | 7.25 | | | 2.54 | | | | 35.0 | | |
| D92 | 2184 | | | | 5 | | | 5.43 | | | 2.05 | | | | 37.8 | | |
| D99 | 2352 | | | | 5 | | | 3.84 | | | 1.65 | | | | 42.9 | | |
| **Total Pozelimab Concentrations** | | | | | | | | | | | | | | | | | |
| **Pozelimab 10 mg/kg + Cemdisiran 25 mg/kg SC** | | | | | | | | | | | | | | | | | |
| **Study Day** | **Time Post Dose (h)** | | | | **n** | | | **Mean** | | | **SD** | | | | **CV** | | |
|  |  |  |  |  |  |  |  | **µg/mL** | | | | | | | **%** | | |
| D1 | 0 | | | | 5 | | | BLQ | | | NC | | | | NC | | |
|  | 0.5 | | | | 5 | | | 0.795 | | | 0.339 | | | | 42.7 | | |
|  | 4 | | | | 5 | | | 21.1 | | | 4.71 | | | | 22.3 | | |
|  | 8 | | | | 5 | | | 40.9 | | | 12.8 | | | | 31.3 | | |
| D2 | 24 | | | | 5 | | | 67.9 | | | 11.7 | | | | 17.2 | | |
| D3 | 48 | | | | 5 | | | 99.7 | | | 10.1 | | | | 10.2 | | |
| D4 | 72 | | | | 5 | | | 107 | | | 9.91 | | | | 9.30 | | |
| D8 | 168 | | | | 5 | | | 98.0 | | | 5.27 | | | | 5.38 | | |
| D11 | 240 | | | | 5 | | | 85.8 | | | 5.16 | | | | 6.02 | | |
| D15 | 336 | | | | | 5 | 74.3 | | | | 7.92 | | | 10.7 | | | |
| D19 | 432 | | | | | 5 | 64.4 | | | | 5.55 | | | 8.62 | | | |
| D22 | 504 | | | | | 5 | 59.6 | | | | 6.73 | | | 11.3 | | | |
| D29 | 672 | | | | | 5 | 48.1 | | | | 7.55 | | | 15.7 | | | |
| D36 | 840 | | | | | 5 | 41.2 | | | | 7.68 | | | 18.6 | | | |
| D43 | 1008 | | | | | 5 | 30.8 | | | | 7.97 | | | 25.9 | | | |
| D50 | 1176 | | | | | 5 | 23.0 | | | | 6.44 | | | 28.1 | | | |
| D57 | 1344 | | | | | 5 | 18.4 | | | | 5.66 | | | 30.8 | | | |
| D64 | 1512 | | | | | 5 | 14.9 | | | | 4.62 | | | 31.0 | | | |
| D71 | 1680 | | | | | 5 | 11.3 | | | | 4.06 | | | 35.9 | | | |
| D78 | 1848 | | | | | 0 | NC | | | | NC | | | NC | | | |
| D85 | 2016 | | | | | 5 | 7.35 | | | | 2.46 | | | 33.4 | | | |
| D92 | 2184 | | | | | 5 | 6.27 | | | | 2.58 | | | 41.1 | | | |
| D99 | 2352 | | | | | 5 | 5.22 | | | | 2.29 | | | 43.9 | | | |

BLQ, below the limit of quantitation; CV, coefficient of variation; NC, not calculated; SD, standard deviation.

Supplementary Table 2. Percent of pre-dose CP hemolysis values

| Cemdisiran (5 mg/kg) | | | | | | | |
| --- | --- | --- | --- | --- | --- | --- | --- |
| Time post-dose (weeks) | **1001** | **1002** | **1003** | **n** | **Mean** | **STDEV** | **%CV** |
| 0 | 106% | 92% | 102% | 3 | 100 | 7.48 | 7.48% |
| 0.02381 | 109% | 92% | 105% | 3 | 102 | 8.63 | 8.46% |
| 0.047619 | 111% | 93% | 100% | 3 | 101 | 9.14 | 9.05% |
| 0.142857 | 99% | 89% | 101% | 3 | 96 | 6.75 | 7.01% |
| 0.285714 | 106% | 101% | 116% | 3 | 107 | 7.77 | 7.24% |
| 0.428571 | 110% | 97% | 108% | 3 | 105 | 7.18 | 6.85% |
| 1 | 99% | 35% | 84% | 3 | 73 | 33.49 | 45.94% |
| 1.428571 | 89% | 27% | 62% | 3 | 59 | 30.84 | 51.96% |
| 2 | 66% | 14% | 59% | 3 | 46 | 28.11 | 60.67% |
| 2.571429 | 83% | 26% | 48% | 3 | 53 | 28.75 | 54.45% |
| 3 | 79% | 17% | 39% | 3 | 45 | 31.35 | 69.33% |
| 4 | 54% | 15% | 23% | 3 | 31 | 20.89 | 68.19% |
| 5 | 47% | 22% | 28% | 3 | 32 | 12.73 | 39.64% |
| 6 | 75% | 20% | 20% | 3 | 38 | 31.82 | 83.27% |
| 7 | 100% | 47% | 30% | 3 | 59 | 36.66 | 61.92% |
| 8 | 106% | 49% | 44% | 3 | 66 | 34.21 | 51.52% |
| 9 | 108% | 78% | 58% | 3 | 81 | 25.36 | 31.21% |
| 10 | 108% | 101% | 61% | 3 | 90 | 25.05 | 27.82% |
| 11 | 112% | 79% | 52% | 3 | 81 | 29.59 | 36.51% |
| 12 | 114% | 92% | 87% | 3 | 98 | 13.99 | 14.29% |
| 13 | 108% | 93% | 83% | 3 | 95 | 12.60 | 13.29% |
| 14 | 111% | 106% | 106% | 3 | 108 | 2.67 | 2.47% |
| 15 | 110% | 112% | 108% | 3 | 110 | 2.21 | 2.00% |
| 16 | 114% | 109% | 109% | 3 | 111 | 3.00 | 2.71% |

| Cemdisiran (25 mg/kg) | | | | | | | |
| --- | --- | --- | --- | --- | --- | --- | --- |
| Time post-dose (weeks) | **2001** | **2002** | **2003** | **n** | **Mean** | **STDEV** | **%CV** |
| 0 | 98% | 101% | 101% |  | 100 | 2.08 | 2.08% |
| 0.02381 | 100% | 109% | 109% | 3 | 106 | 5.25 | 4.95% |
| 0.047619 | 100% | 110% | 95% | 3 | 102 | 7.91 | 7.78% |
| 0.142857 | 109% | 103% | 116% | 3 | 110 | 6.48 | 5.91% |
| 0.285714 | 94% | 105% | 110% | 3 | 103 | 8.45 | 8.21% |
| 0.428571 | 102% | 107% | 105% | 3 | 105 | 2.29 | 2.19% |
| 1 | 37% | 84% | 65% | 3 | 62 | 23.75 | 38.19% |
| 1.428571 | 14% | 27% | 22% | 3 | 21 | 6.94 | 33.29% |
| 2 | 9% | 27% | 18% | 3 | 18 | 8.82 | 48.76% |
| 2.571429 | 15% | 26% | 27% | 3 | 23 | 6.37 | 28.16% |
| 3 | 15% | 20% | 12% | 3 | 16 | 4.13 | 25.81% |
| 4 | 16% | 10% | 8% | 3 | 12 | 3.84 | 33.20% |
| 5 | 6% | 9% | 9% | 3 | 8 | 1.84 | 23.34% |
| 6 | 7% | 11% | 19% | 3 | 12 | 6.07 | 50.02% |
| 7 | 13% | 17% | 22% | 3 | 17 | 4.53 | 26.57% |
| 8 | 14% | 29% | 30% | 3 | 24 | 8.84 | 36.76% |
| 9 | 15% | 24% | 41% | 3 | 27 | 13.45 | 50.22% |
| 10 | 15% | 25% | 45% | 3 | 29 | 15.28 | 53.46% |
| 11 | 11% | 31% | 76% | 3 | 39 | 33.51 | 85.22% |
| 12 | 14% | 38% | 67% | 3 | 40 | 26.82 | 67.43% |
| 13 | 31% | 52% | 96% | 3 | 60 | 32.91 | 55.15% |
| 14 | 31% | 77% | 107% | 3 | 72 | 37.88 | 52.74% |
| 15 | 23% | 76% | 109% | 3 | 69 | 43.15 | 62.33% |
| 16 | 36% | 102% | 118% | 3 | 85 | 43.26 | 50.75% |

| Pozelimab (5 mg/kg) | | | | | | | | |
| --- | --- | --- | --- | --- | --- | --- | --- | --- |
| Time post-dose (weeks) | **3001** | **3002** | **3003** | **3004** | **n** | **Mean** | **STDEV** | **%CV** |
| 0 | 98% | 100% | 100% | 101% | 4 | 100 | 1.33 | 1.33% |
| 0.002976 | 81% | 105% | 98% | 97% | 4 | 95 | 10.09 | 10.59% |
| 0.02381 | 78% | 102% | 92% | 91% | 4 | 91 | 9.74 | 10.73% |
| 0.047619 | 61% | 80% | 39% | 77% | 4 | 64 | 18.75 | 29.28% |
| 0.142857 | 16% | 44% | 23% | 27% | 4 | 27 | 11.72 | 42.70% |
| 0.285714 | 39% | 18% | 14% | 23% | 4 | 23 | 10.76 | 45.89% |
| 0.428571 | 20% | 43% | 17% | 29% | 4 | 27 | 11.72 | 43.17% |
| 1 | 19% | 25% | 14% | 25% | 4 | 21 | 5.36 | 26.14% |
| 1.428571 | 43% | 21% | 10% | 18% | 4 | 23 | 14.11 | 61.23% |
| 2 | 42% | 45% | 13% | 77% | 4 | 44 | 25.98 | 58.78% |
| 2.571429 | 50% | 58% | 27% | 96% | 4 | 58 | 28.93 | 50.05% |
| 3 | 42% | 98% | 29% | 88% | 4 | 64 | 33.57 | 52.28% |
| 4 | 83% | 109% | 74% | 112% | 4 | 94 | 18.79 | 19.92% |
| 5 | 93% | 106% | 85% | 109% | 4 | 98 | 11.21 | 11.42% |
| 6 | 103% | 114% | 113% | 116% | 4 | 111 | 5.64 | 5.06% |
| 7 | 86% | 98% | 100% | 101% | 4 | 96 | 7.08 | 7.36% |
| 8 | 103% | 112% | 115% | 116% | 4 | 112 | 5.71 | 5.12% |
| 9 | 91% | 106% | 107% | 109% | 4 | 103 | 8.15 | 7.91% |
| 10 | 114% | 106% | 106% | 110% | 4 | 109 | 3.94 | 3.61% |

| Pozelimab (10 mg/kg) | | | | | | | | |
| --- | --- | --- | --- | --- | --- | --- | --- | --- |
| Time post-dose (weeks) | **4001** | **4002** | **4003** | **4004** | **n** | **Mean** | **STDEV** | **%CV** |
| 0 | 99% | 98% | 100% | 103% | 4 | 100 | 2.09 | 2.09% |
| 0.002976 | 92% | 87% | 91% | 94% | 4 | 91 | 2.96 | 3.24% |
| 0.02381 | 12% | 26% | 92% | 67% | 4 | 49 | 36.82 | 74.56% |
| 0.047619 | 10% | 19% | 12% | 11% | 4 | 13 | 3.98 | 30.81% |
| 0.142857 | 10% | 7% | 12% | 11% | 4 | 10 | 1.86 | 18.78% |
| 0.285714 | 8% | 10% | 14% | 14% | 4 | 12 | 2.82 | 24.28% |
| 0.428571 | 11% | 20% | 24% | 24% | 4 | 20 | 6.44 | 32.71% |
| 1 | 7% | 7% | 9% | 8% | 4 | 8 | 1.14 | 14.91% |
| 1.428571 | 7% | 8% | 10% | 13% | 4 | 9 | 2.79 | 29.84% |
| 2 | 8% | 12% | 34% | 22% | 4 | 19 | 11.88 | 62.64% |
| 2.571429 | 28% | 22% | 37% | 20% | 4 | 27 | 7.51 | 28.29% |
| 3 | 14% | 16% | 41% | 24% | 4 | 24 | 12.19 | 50.96% |
| 4 | 26% | 20% | 96% | 57% | 4 | 50 | 34.75 | 69.67% |
| 5 | 53% | 49% | 100% | 73% | 4 | 69 | 23.61 | 34.36% |
| 6 | 90% | 69% | 109% | 103% | 4 | 93 | 18.01 | 19.43% |
| 7 | 91% | 94% | 98% | 102% | 4 | 96 | 5.05 | 5.26% |
| 8 | 109% | 109% | 113% | 100% | 4 | 108 | 5.39 | 4.99% |
| 9 | 111% | 112% | 110% | 109% | 4 | 111 | 1.53 | 1.38% |
| 10 | 103% | 102% | 109% | 108% | 4 | 105 | 3.62 | 3.44% |

| Cemdisiran (5 mg/kg) + Pozelimab (5 mg/kg) | | | | | | | | | |
| --- | --- | --- | --- | --- | --- | --- | --- | --- | --- |
| Time post-dose (weeks) | **5001** | **5002** | **5003** | **5004** | **5005** | **n** | **Mean** | **STDEV** | **%CV** |
| 0 | 102% | 96% | 106% | 101% | 95% | 5 | 100 | 4.42 | 4.42% |
| 2 | 58% | 69% | 102% | 28% | 64% | 5 | 64 | 26.50 | 41.19% |
| 2.002976 | 36% | 43% | 50% | 16% | 82% | 5 | 45 | 24.28 | 53.43% |
| 2.02381 | 7% | 15% | 9% | 7% | 4% | 5 | 8 | 4.25 | 50.48% |
| 2.047619 | 5% | 4% | 3% | 1% | 2% | 5 | 3 | 1.61 | 53.44% |
| 2.142857 | 4% | 5% | 5% | 4% | 4% | 5 | 4 | 0.74 | 16.85% |
| 2.285714 | 4% | 8% | 4% | 1% | 4% | 5 | 4 | 2.52 | 58.00% |
| 2.428571 | 5% | 8% | 7% | 4% | 5% | 5 | 6 | 1.73 | 29.52% |
| 3 | 3% | 3% | 4% | 4% | 2% | 5 | 3 | 0.77 | 24.46% |
| 3.428571 | 3% | 5% | 6% | 4% | 2% | 5 | 4 | 1.46 | 38.34% |
| 4 | 5% | 4% | 4% | 3% | 3% | 5 | 4 | 1.12 | 28.18% |
| 4.571429 | 5% | 6% | 7% | 4% | 4% | 5 | 5 | 1.23 | 22.77% |
| 5 | 4% | 5% | 4% | 3% | 3% | 5 | 4 | 0.72 | 19.27% |
| 6 | 6% | 6% | 6% | 3% | 5% | 5 | 5 | 1.42 | 28.06% |
| 7 | 7% | 9% | 11% | 6% | 10% | 5 | 9 | 2.00 | 22.78% |
| 8 | 6% | 10% | 15% | 8% | 13% | 5 | 10 | 3.41 | 32.90% |
| 9 | 6% | 17% | 40% | 20% | 10% | 5 | 19 | 13.29 | 71.29% |
| 10 | 9% | 18% | 28% | 6% | 17% | 5 | 15 | 8.75 | 57.14% |
| 11 | 30% | 67% | 97% | 29% | 81% | 5 | 61 | 30.35 | 49.84% |
| 12 | 45% | 93% | 95% | 25% | 62% | 5 | 64 | 30.27 | 47.11% |
| 13 | 98% | 105% | 107% | 70% | 105% | 5 | 97 | 15.67 | 16.18% |
| 14 | 102% | 109% | 110% | 56% | 95% | 5 | 94 | 22.12 | 23.47% |
| 15 | 89% | 101% | 101% | 57% | 96% | 5 | 89 | 18.56 | 20.94% |
| 16 | 110% | 113% | 114% | 80% | 98% | 5 | 103 | 14.16 | 13.76% |

| Cemdisiran (5 mg/kg) + Pozelimab (10 mg/kg) | | | | | | | | | |
| --- | --- | --- | --- | --- | --- | --- | --- | --- | --- |
| Time post-dose (weeks) | **6001** | **6002** | **6003** | **6004** | **6005** | **n** | **Mean** | **STDEV** | **%CV** |
| 0 | 109% | 94% | 96% | 103% | 98% | 5 | 100 | 5.71 | 5.71% |
| 2 | 47% | 38% | 63% | 123% | 18% | 5 | 58 | 40.01 | 69.06% |
| 2.002976 | 53% | 29% | 45% | 103% | 29% | 5 | 52 | 30.78 | 59.50% |
| 2.02381 | 8% | 2% | 3% | 11% | 2% | 5 | 5 | 4.34 | 82.26% |
| 2.047619 | 2% | 2% | 2% | 7% | 2% | 5 | 3 | 2.13 | 69.61% |
| 2.142857 | 4% | 3% | 4% | 5% | 1% | 5 | 3 | 1.43 | 41.76% |
| 2.285714 | 2% | 2% | 3% | 3% | 3% | 5 | 3 | 0.54 | 20.78% |
| 2.428571 | 3% | 2% | 2% | 11% | 2% | 5 | 4 | 3.75 | 94.09% |
| 3 | 3% | 2% | 2% | 2% | 2% | 5 | 2 | 0.48 | 21.86% |
| 3.428571 | 3% | 3% | 3% | 2% | 1% | 5 | 3 | 0.82 | 32.22% |
| 4 | 3% | 4% | 2% | 8% | 2% | 5 | 4 | 2.53 | 69.81% |
| 4.571429 | 4% | 4% | 6% | 9% | 3% | 5 | 5 | 2.46 | 47.63% |
| 5 | 3% | 3% | 3% | 8% | 2% | 5 | 4 | 2.35 | 63.57% |
| 6 | 3% | 2% | 2% | 8% | 3% | 5 | 4 | 2.61 | 71.23% |
| 7 | 6% | 6% | 5% | 9% | 3% | 5 | 6 | 2.48 | 43.95% |
| 8 | 6% | 3% | 2% | 8% | 3% | 5 | 4 | 2.50 | 56.21% |
| 9 | 12% | 8% | 3% | 37% | 8% | 5 | 14 | 13.24 | 96.78% |
| 10 | 11% | 4% | 6% | 42% | 9% | 5 | 14 | 15.70 | 109.89% |
| 11 | 87% | 8% | 9% | 90% | 57% | 5 | 50 | 40.11 | 80.08% |
| 12 | 63% | 18% | 14% | 89% | 90% | 5 | 55 | 37.13 | 67.97% |
| 13 | 111% | 39% | 22% | 112% | 97% | 5 | 76 | 42.77 | 56.10% |
| 14 | 102% | 39% | 37% | 104% | 78% | 5 | 72 | 32.84 | 45.70% |
| 15 | 96% | 55% | 67% | 114% | 103% | 5 | 87 | 24.92 | 28.69% |
| 16 | 102% | 93% | 95% | 105% | 97% | 5 | 98 | 5.08 | 5.17% |

| Cemdisiran (25 mg/kg) + Pozelimab (10 mg/kg) | | | | | | | | | |
| --- | --- | --- | --- | --- | --- | --- | --- | --- | --- |
| Time post-dose (weeks) | **7001** | **7002** | **7003** | **7004** | **7005** | **n** | **Mean** | **STDEV** | **%CV** |
| 0 | 99% | 100% | 106% | 95% | 99% | 5 | 100 | 4.00 | 4.00% |
| 2 | 7% | 8% | 37% | 45% | 15% | 5 | 22 | 17.26 | 77.87% |
| 2.002976 | 16% | 12% | 55% | 79% | 5% | 5 | 33 | 32.01 | 96.19% |
| 2.02381 | 2% | 2% | 4% | 3% | 2% | 5 | 2 | 0.82 | 33.27% |
| 2.047619 | 1% | 2% | 1% | 2% | 1% | 5 | 2 | 0.15 | 9.78% |
| 2.142857 | 2% | 3% | 3% | 5% | 2% | 5 | 3 | 1.12 | 36.93% |
| 2.285714 | 2% | 1% | 3% | 3% | 4% | 5 | 3 | 0.92 | 35.69% |
| 2.428571 | 4% | 1% | 2% | 1% | 2% | 5 | 2 | 1.09 | 48.67% |
| 3 | 2% | 3% | 4% | 5% | 1% | 5 | 3 | 1.36 | 46.76% |
| 3.428571 | 5% | 2% | 4% | 3% | 3% | 5 | 3 | 1.07 | 32.84% |
| 4 | 3% | 2% | 3% | 2% | 3% | 5 | 3 | 0.47 | 18.51% |
| 4.571429 | 4% | 4% | 2% | 3% | 8% | 5 | 4 | 2.29 | 56.55% |
| 5 | 2% | 2% | 2% | 4% | 2% | 5 | 2 | 0.74 | 29.88% |
| 6 | 3% | 1% | 3% | 3% | 2% | 5 | 3 | 0.85 | 33.74% |
| 7 | 4% | 4% | 4% | 3% | 4% | 5 | 4 | 0.63 | 15.97% |
| 8 | 3% | 7% | 3% | 2% | 4% | 5 | 4 | 2.04 | 50.92% |
| 9 | 3% | 2% | 4% | 2% | 2% | 5 | 2 | 0.93 | 37.63% |
| 10 | 3% | 2% | 4% | 4% | 2% | 5 | 3 | 0.96 | 29.91% |
| 11 | 8% | 4% | 6% | 3% | 4% | 5 | 5 | 2.02 | 39.56% |
| 12 | 7% | 5% | 11% | 5% | 4% | 5 | 6 | 2.68 | 43.93% |
| 13 | 9% | 5% | 14% | 7% | 4% | 5 | 8 | 3.82 | 49.43% |
| 14 | 25% | 9% | 26% | 8% | 5% | 5 | 15 | 10.08 | 68.45% |
| 15 | 40% | 11% | 22% | 9% | 10% | 5 | 18 | 13.33 | 73.40% |
| 16 | 80% | 33% | 55% | 25% | 26% | 5 | 44 | 23.64 | 54.29% |

CV, coefficient of variation; STDEV, standard deviation.

Supplementary Table 3. Total C5 concentration (µg/mL) by LC-MS according to treatment group

| **Cemdisiran (5 mg/kg)** | | | | | | | | | | | | | | | | | | | | | | | | | | | | | | | | | | | | | | | | |  |
| --- | --- | --- | --- | --- | --- | --- | --- | --- | --- | --- | --- | --- | --- | --- | --- | --- | --- | --- | --- | --- | --- | --- | --- | --- | --- | --- | --- | --- | --- | --- | --- | --- | --- | --- | --- | --- | --- | --- | --- | --- | --- |
| **Time post-dose (weeks)** | **1001** | | | | **1002** | | | | | | **1003** | | | | | **n** | | | **Mean** | | | | | **STDEV** | | | | | | | | | **%CV** | | | | | | | |  |
| 0 | 74.2 | | | | 54.8 | | | | | | 90.1 | | | | | 3 | | | 73.0 | | | | | 17.7 | | | | | | | | | 24.2% | | | | | | | |  |
| 0.02381 | 113 | | | | 89.3 | | | | | | 156 | | | | | 3 | | | 119 | | | | | 34 | | | | | | | | | 28.2% | | | | | | | |  |
| 0.047619 | 109 | | | | 82.9 | | | | | | 138 | | | | | 3 | | | 110 | | | | | 27 | | | | | | | | | 24.9% | | | | | | | |  |
| 0.142857 | 98.3 | | | | 81.8 | | | | | | 141 | | | | | 3 | | | 106.9 | | | | | 30.3 | | | | | | | | | 28.4% | | | | | | | |  |
| 0.285714 | 93.6 | | | | 60.9 | | | | | | 115 | | | | | 3 | | | 89.7 | | | | | 27.1 | | | | | | | | | 30.2% | | | | | | | |  |
| 0.428571 | 76.0 | | | | 44.6 | | | | | | 87.7 | | | | | 3 | | | 69.4 | | | | | 22.3 | | | | | | | | | 32.1% | | | | | | | |  |
| 1 | 36.9 | | | | 20.9 | | | | | | 46.3 | | | | | 3 | | | 34.7 | | | | | 12.8 | | | | | | | | | 36.9% | | | | | | | |  |
| 1.428571 | 28.8 | | | | 15.5 | | | | | | 31.3 | | | | | 3 | | | 25.2 | | | | | 8.5 | | | | | | | | | 33.6% | | | | | | | |  |
| 2 | 21.8 | | | | 10.7 | | | | | | 18.1 | | | | | 3 | | | 16.9 | | | | | 5.7 | | | | | | | | | 33.5% | | | | | | | |  |
| 2.571429 | 19.4 | | | | 9.5 | | | | | | 15.5 | | | | | 3 | | | 14.8 | | | | | 5.0 | | | | | | | | | 33.6% | | | | | | | |  |
| 3 | 19.0 | | | | 7.5 | | | | | | 11.4 | | | | | 3 | | | 12.6 | | | | | 5.9 | | | | | | | | | 46.5% | | | | | | | |  |
| 4 | 20.8 | | | | 8.3 | | | | | | 10.1 | | | | | 3 | | | 13.1 | | | | | 6.8 | | | | | | | | | 51.6% | | | | | | | |  |
| 5 | 24.3 | | | | 11.2 | | | | | | 11.6 | | | | | 3 | | | 15.7 | | | | | 7.4 | | | | | | | | | 47.2% | | | | | | | |  |
| 6 | 29.9 | | | | 13.2 | | | | | | 12.9 | | | | | 3 | | | 18.6 | | | | | 9.7 | | | | | | | | | 52.1% | | | | | | | |  |
| 7 | 36.1 | | | | 17.8 | | | | | | 13.4 | | | | | 3 | | | 22.4 | | | | | 12.0 | | | | | | | | | 53.4% | | | | | | | |  |
| 8 | 47.1 | | | | 23.0 | | | | | | 16.8 | | | | | 3 | | | 29.0 | | | | | 16.0 | | | | | | | | | 55.2% | | | | | | | |  |
| 9 | 60.6 | | | | 28.9 | | | | | | 20.5 | | | | | 3 | | | 36.7 | | | | | 21.1 | | | | | | | | | 57.6% | | | | | | | |  |
| 10 | 81.5 | | | | 43.4 | | | | | | 25.5 | | | | | 3 | | | 50.2 | | | | | 28.6 | | | | | | | | | 56.9% | | | | | | | |  |
| 11 | 66.2 | | | | 53.6 | | | | | | 31.8 | | | | | 3 | | | 50.5 | | | | | 17.4 | | | | | | | | | 34.5% | | | | | | | |  |
| 12 | 62.8 | | | | 49.9 | | | | | | 32.2 | | | | | 3 | | | 48.3 | | | | | 15.3 | | | | | | | | | 31.7% | | | | | | | |  |
| 13 | 64.0 | | | | 46.0 | | | | | | 34.4 | | | | | 3 | | | 48.1 | | | | | 14.9 | | | | | | | | | 31.0% | | | | | | | |  |
| 14 | 83.7 | | | | 49.6 | | | | | | 53.7 | | | | | 3 | | | 62.4 | | | | | 18.6 | | | | | | | | | 29.8% | | | | | | | |  |
| 15 | 66.6 | | | | 58.8 | | | | | | 39.8 | | | | | 3 | | | 55.1 | | | | | 13.8 | | | | | | | | | 25.0% | | | | | | | |  |
| 16 | 77.7 | | | | 55.2 | | | | | | 45.5 | | | | | 3 | | | 59.5 | | | | | 16.5 | | | | | | | | | 27.8% | | | | | | | |  |
| **Cemdisiran (25 mg/kg)** | | | | | | | | | | | | | | | | | | | | | | | | | | | | | | | | | | | | | | | | |  |
| **Time post-dose (weeks)** | | **2001** | | | | | **2002** | | | | | | **2003** | | | | | **n** | | **Mean** | | | | | **STDEV** | | | | | | | | | **%CV** | | | | | | |  |
| 0 | | 40.3 | | | | | 74.8 | | | | | | 73.0 | | | | | 3 | | 62.7 | | | | | 19.4 | | | | | | | | | 31.0% | | | | | | |  |
| 0.02381 | | 53 | | | | | 129 | | | | | | 137 | | | | | 3 | | 106 | | | | | 47 | | | | | | | | | 43.9% | | | | | | |  |
| 0.047619 | | 59 | | | | | 110 | | | | | | 115 | | | | | 3 | | 95 | | | | | 31 | | | | | | | | | 32.6% | | | | | | |  |
| 0.142857 | | 52.9 | | | | | 98.2 | | | | | | 109 | | | | | 3 | | 86.7 | | | | | 29.7 | | | | | | | | | 34.3% | | | | | | |  |
| 0.285714 | | 47.2 | | | | | 80.4 | | | | | | 86 | | | | | 3 | | 71.3 | | | | | 21.0 | | | | | | | | | 29.5% | | | | | | |  |
| 0.428571 | | 29.6 | | | | | 53.0 | | | | | | 62.6 | | | | | 3 | | 48.4 | | | | | 17.0 | | | | | | | | | 35.0% | | | | | | |  |
| 1 | | 9.3 | | | | | 20.2 | | | | | | 23.3 | | | | | 3 | | 17.6 | | | | | 7.4 | | | | | | | | | 41.8% | | | | | | |  |
| 1.428571 | | 6.0 | | | | | 13.7 | | | | | | 14.1 | | | | | 3 | | 11.3 | | | | | 4.6 | | | | | | | | | 40.7% | | | | | | |  |
| 2 | | 2.9 | | | | | 8.1 | | | | | | 7.5 | | | | | 3 | | 6.2 | | | | | 2.8 | | | | | | | | | 46.0% | | | | | | |  |
| 2.571429 | | 2.5 | | | | | 6.0 | | | | | | 7.0 | | | | | 3 | | 5.2 | | | | | 2.4 | | | | | | | | | 45.7% | | | | | | |  |
| 3 | | 1.9 | | | | | 4.6 | | | | | | 4.6 | | | | | 3 | | 3.7 | | | | | 1.6 | | | | | | | | | 42.4% | | | | | | |  |
| 4 | | 1.6 | | | | | 4.6 | | | | | | 4.1 | | | | | 3 | | 3.4 | | | | | 1.6 | | | | | | | | | 48.1% | | | | | | |  |
| 5 | | 1.7 | | | | | 4.3 | | | | | | 4.0 | | | | | 3 | | 3.3 | | | | | 1.4 | | | | | | | | | 43.4% | | | | | | |  |
| 6 | | 2.1 | | | | | 4.3 | | | | | | 5.1 | | | | | 3 | | 3.9 | | | | | 1.6 | | | | | | | | | 40.2% | | | | | | |  |
| 7 | | 2.1 | | | | | 4.8 | | | | | | 5.9 | | | | | 3 | | 4.3 | | | | | 2.0 | | | | | | | | | 46.2% | | | | | | |  |
| 8 | | 2.0 | | | | | 6.4 | | | | | | 6.8 | | | | | 3 | | 5.1 | | | | | 2.6 | | | | | | | | | 52.1% | | | | | | |  |
| 9 | | 2.3 | | | | | 7.1 | | | | | | 9.4 | | | | | 3 | | 6.3 | | | | | 3.6 | | | | | | | | | 57.5% | | | | | | |  |
| 10 | | 2.8 | | | | | 8.8 | | | | | | 15.0 | | | | | 3 | | 8.9 | | | | | 6.1 | | | | | | | | | 69.3% | | | | | | |  |
| 11 | | 3.9 | | | | | 10.6 | | | | | | 21.0 | | | | | 3 | | 11.8 | | | | | 8.6 | | | | | | | | | 72.6% | | | | | | |  |
| 12 | | 3.4 | | | | | 9.9 | | | | | | 19.2 | | | | | 3 | | 10.9 | | | | | 7.9 | | | | | | | | | 73.1% | | | | | | |  |
| 13 | | 4.3 | | | | | 11.4 | | | | | | 23.5 | | | | | 3 | | 13.1 | | | | | 9.7 | | | | | | | | | 74.2% | | | | | | |  |
| 14 | | 4.5 | | | | | 12.1 | | | | | | 31.4 | | | | | 3 | | 16.0 | | | | | 13.9 | | | | | | | | | 86.7% | | | | | | |  |
| 15 | | 4.0 | | | | | 13.9 | | | | | | 30.1 | | | | | 3 | | 16.0 | | | | | 13.2 | | | | | | | | | 82.3% | | | | | | |  |
| 16 | | 4.7 | | | | | 16.5 | | | | | | 36.2 | | | | | 3 | | 19.1 | | | | | 15.9 | | | | | | | | | 83.2% | | | | | | |  |
| **Pozelimab (5 mg/kg)** | | | | | | | | | | | | | | | | | | | | | | | | | | | | | | | | | | | | | | | | |  |
| **Time post-dose (weeks)** | **3001** | | | **3002** | | | | | **3003** | | | | | | **3004** | | | | | | | **n** | | | **Mean** | | | | | | **STDEV** | | | | | | **%CV** | | | |  |
| 0 | 76.8 | | | 69.0 | | | | | 59.8 | | | | | | 68.5 | | | | | | | 4 | | | 68.5 | | | | | | 6.9 | | | | | | 10.1% | | | |  |
| 0.002976 | 81.9 | | | 73.1 | | | | | 66.6 | | | | | | 73.8 | | | | | | | 4 | | | 74 | | | | | | 6 | | | | | | 8.5% | | | |  |
| 0.02381 | 128 | | | 106 | | | | | 85 | | | | | | 106 | | | | | | | 4 | | | 106 | | | | | | 18 | | | | | | 16.7% | | | |  |
| 0.047619 | 118 | | | 100 | | | | | 92 | | | | | | 104 | | | | | | | 4 | | | 103.7 | | | | | | 10.9 | | | | | | 10.6% | | | |  |
| 0.142857 | 133 | | | 113 | | | | | 103 | | | | | | 116 | | | | | | | 4 | | | 116.4 | | | | | | 12.3 | | | | | | 10.5% | | | |  |
| 0.285714 | 161 | | | 181 | | | | | 115 | | | | | | 152 | | | | | | | 4 | | | 152.2 | | | | | | 27.6 | | | | | | 18.1% | | | |  |
| 0.428571 | 160 | | | 147 | | | | | 117 | | | | | | 141 | | | | | | | 4 | | | 141.3 | | | | | | 18.0 | | | | | | 12.7% | | | |  |
| 1 | 160 | | | 131 | | | | | 126 | | | | | | 139 | | | | | | | 4 | | | 139.0 | | | | | | 14.8 | | | | | | 10.7% | | | |  |
| 1.428571 | 196 | | | 143 | | | | | 127 | | | | | | 155 | | | | | | | 4 | | | 155.4 | | | | | | 29.7 | | | | | | 19.1% | | | |  |
| 2 | 178 | | | 139 | | | | | 123 | | | | | | 147 | | | | | | | 4 | | | 146.7 | | | | | | 23.0 | | | | | | 15.6% | | | |  |
| 2.571429 | 163 | | | 128 | | | | | 113 | | | | | | 135 | | | | | | | 4 | | | 134.8 | | | | | | 20.8 | | | | | | 15.5% | | | |  |
| 3 | 139 | | | 127 | | | | | 109 | | | | | | 125 | | | | | | | 4 | | | 125.1 | | | | | | 12.1 | | | | | | 9.7% | | | |  |
| 4 | 128 | | | 110 | | | | | 109 | | | | | | 116 | | | | | | | 4 | | | 115.7 | | | | | | 9.1 | | | | | | 7.8% | | | |  |
| 5 | 115 | | | 105 | | | | | 102 | | | | | | 107 | | | | | | | 4 | | | 107.4 | | | | | | 5.7 | | | | | | 5.3% | | | |  |
| 6 | 125 | | | 133 | | | | | 103 | | | | | | 120 | | | | | | | 4 | | | 120.3 | | | | | | 12.9 | | | | | | 10.7% | | | |  |
| 7 | 120 | | | 101 | | | | | 101 | | | | | | 107 | | | | | | | 4 | | | 107.1 | | | | | | 9.3 | | | | | | 8.7% | | | |  |
| 8 | 118 | | | 112 | | | | | 98 | | | | | | 109 | | | | | | | 4 | | | 109.3 | | | | | | 8.6 | | | | | | 7.8% | | | |  |
| 9 | 104 | | | 92 | | | | | 125 | | | | | | 107 | | | | | | | 4 | | | 106.7 | | | | | | 13.7 | | | | | | 12.9% | | | |  |
| 10 | 153 | | | 134 | | | | | 103 | | | | | | 130 | | | | | | | 4 | | | 129.7 | | | | | | 20.5 | | | | | | 15.8% | | | |  |
| **Pozelimab (10 mg/kg)** | | | | | | | | | | | | | | | | | | | | | | | | | | | | | | | | | | | | | | | | |  |
| **Time post-dose (weeks)** | **4001** | | | | | | **4002** | | | | | **4003** | | | | | **4004** | | | | | | **n** | | | | **Mean** | | | | | **STDEV** | | | | | | **%CV** | | |  |
| 0 | 65.2 | | | | | | 65.6 | | | | | 122.0 | | | | | 80.3 | | | | | | 4 | | | | 83.3 | | | | | 26.8 | | | | | | 32.1% | | |  |
| 0.002976 | 118.0 | | | | | | 82.9 | | | | | 116.2 | | | | | 136.0 | | | | | | 4 | | | | 113.3 | | | | | 22.1 | | | | | | 19.5% | | |  |
| 0.02381 | 106 | | | | | | 88 | | | | | 154 | | | | | 119 | | | | | | 4 | | | | 117 | | | | | 28.1 | | | | | | 24.1% | | |  |
| 0.047619 | 103 | | | | | | 93 | | | | | 162 | | | | | 111 | | | | | | 4 | | | | 117 | | | | | 30.9 | | | | | | 26.3% | | |  |
| 0.142857 | 98 | | | | | | 118 | | | | | 179 | | | | | 122 | | | | | | 4 | | | | 129 | | | | | 34.6 | | | | | | 26.8% | | |  |
| 0.285714 | 148 | | | | | | 130 | | | | | 225 | | | | | 167 | | | | | | 4 | | | | 167 | | | | | 41.1 | | | | | | 24.6% | | |  |
| 0.428571 | 173 | | | | | | 142 | | | | | 226 | | | | | 159 | | | | | | 4 | | | | 175 | | | | | 36.2 | | | | | | 20.7% | | |  |
| 1 | 157 | | | | | | 117 | | | | | 227 | | | | | 151 | | | | | | 4 | | | | 163 | | | | | 46.0 | | | | | | 28.2% | | |  |
| 1.428571 | 160 | | | | | | 134 | | | | | 235 | | | | | 180 | | | | | | 4 | | | | 177 | | | | | 43.0 | | | | | | 24.2% | | |  |
| 2 | 163 | | | | | | 120 | | | | | 285 | | | | | 180 | | | | | | 4 | | | | 187 | | | | | 69.8 | | | | | | 37.3% | | |  |
| 2.571429 | 166 | | | | | | 120 | | | | | 243 | | | | | 182 | | | | | | 4 | | | | 178 | | | | | 50.7 | | | | | | 28.5% | | |  |
| 3 | 160 | | | | | | 119 | | | | | 265 | | | | | 197 | | | | | | 4 | | | | 185 | | | | | 61.9 | | | | | | 33.4% | | |  |
| 4 | 162 | | | | | | 106 | | | | | 246 | | | | | 202 | | | | | | 4 | | | | 179 | | | | | 59.3 | | | | | | 33.1% | | |  |
| 5 | 155 | | | | | | 105 | | | | | 196 | | | | | 196 | | | | | | 4 | | | | 163 | | | | | 43.3 | | | | | | 26.5% | | |  |
| 6 | 125 | | | | | | 113 | | | | | 160 | | | | | 191 | | | | | | 4 | | | | 147 | | | | | 35.1 | | | | | | 23.9% | | |  |
| 7 | 129 | | | | | | 114 | | | | | 137 | | | | | 186 | | | | | | 4 | | | | 141 | | | | | 31.1 | | | | | | 22.0% | | |  |
| 8 | 144 | | | | | | 98 | | | | | 166 | | | | | 209 | | | | | | 4 | | | | 154 | | | | | 46.5 | | | | | | 30.1% | | |  |
| 9 | 92 | | | | | | 82 | | | | | 139 | | | | | 154 | | | | | | 4 | | | | 117 | | | | | 35.1 | | | | | | 30.0% | | |  |
| 10 | 98 | | | | | | 101 | | | | | 173 | | | | | 175 | | | | | | 4 | | | | 137 | | | | | 43.3 | | | | | | 31.6% | | |  |
| **Cemdisiran (5 mg/kg) + Pozelimab (5 mg/kg)** | | | | | | | | | | | | | | | | | | | | | | | | | | | | | | | | | | | | | | | | | |
| **Time post-dose (weeks)** | **5001** | | | **5002** | | | | | **5003** | | | | | | **5004** | | | | | | | **5005** | | | | | | **n** | | **Mean** | | | | | | **STDEV** | | | | | **%CV** |
| 0 | 88.6 | | | 75.3 | | | | | 43.5 | | | | | | 51.1 | | | | | | | 77.3 | | | | | | 5 | | 67.2 | | | | | | 19.0 | | | | | 28.3% |
| 2 | 15.9 | | | 12.1 | | | | | 20.5 | | | | | | 9.5 | | | | | | | 14.0 | | | | | | 5 | | 14.4 | | | | | | 4.2 | | | | | 29.1% |
| 2.002976 | 18.4 | | | 14.2 | | | | | 43.1 | | | | | | 10.7 | | | | | | | 13.1 | | | | | | 5 | | 19.9 | | | | | | 13.3 | | | | | 66.8% |
| 2.02381 | 21.2 | | | 17.0 | | | | | 29.6 | | | | | | 11.9 | | | | | | | 17.8 | | | | | | 5 | | 19.5 | | | | | | 6.6 | | | | | 33.7% |
| 2.047619 | 21.9 | | | 15.2 | | | | | 30.4 | | | | | | 11.1 | | | | | | | 17.0 | | | | | | 5 | | 19.1 | | | | | | 7.4 | | | | | 38.6% |
| 2.142857 | 20.4 | | | 17.0 | | | | | 32.7 | | | | | | 13.4 | | | | | | | 18.6 | | | | | | 5 | | 20.4 | | | | | | 7.4 | | | | | 36.0% |
| 2.285714 | 23.7 | | | 20.4 | | | | | 37.3 | | | | | | 17.0 | | | | | | | 21.2 | | | | | | 5 | | 23.9 | | | | | | 7.8 | | | | | 32.8% |
| 2.428571 | 19.5 | | | 15.9 | | | | | 33.9 | | | | | | 17.6 | | | | | | | 20.0 | | | | | | 5 | | 21.4 | | | | | | 7.2 | | | | | 33.6% |
| 3 | 20.9 | | | 13.8 | | | | | 32.3 | | | | | | 14.9 | | | | | | | 18.4 | | | | | | 5 | | 20.1 | | | | | | 7.4 | | | | | 36.9% |
| 3.428571 | 20.5 | | | 16.1 | | | | | 30.9 | | | | | | 13.4 | | | | | | | 18.7 | | | | | | 5 | | 19.9 | | | | | | 6.7 | | | | | 33.6% |
| 4 | 19.8 | | | 17.8 | | | | | 31.5 | | | | | | 14.8 | | | | | | | 19.6 | | | | | | 5 | | 20.7 | | | | | | 6.4 | | | | | 30.8% |
| 4.571429 | 21.1 | | | 16.4 | | | | | 35.3 | | | | | | 13.6 | | | | | | | 22.2 | | | | | | 5 | | 21.7 | | | | | | 8.4 | | | | | 38.5% |
| 5 | 19.8 | | | 15.6 | | | | | 30.0 | | | | | | 14.7 | | | | | | | 19.5 | | | | | | 5 | | 19.9 | | | | | | 6.1 | | | | | 30.4% |
| 6 | 21.8 | | | 17.6 | | | | | 32.9 | | | | | | 14.5 | | | | | | | 25.7 | | | | | | 5 | | 22.5 | | | | | | 7.2 | | | | | 31.9% |
| 7 | 27.1 | | | 21.1 | | | | | 41.7 | | | | | | 19.1 | | | | | | | 31.3 | | | | | | 5 | | 28.0 | | | | | | 9.0 | | | | | 32.1% |
| 8 | 30.6 | | | 24.9 | | | | | 44.9 | | | | | | 19.3 | | | | | | | 37.5 | | | | | | 5 | | 31.4 | | | | | | 10.1 | | | | | 32.1% |
| 9 | 38.3 | | | 34.1 | | | | | 55.4 | | | | | | 46.3 | | | | | | | 22.1 | | | | | | 5 | | 39.3 | | | | | | 12.6 | | | | | 32.0% |
| 10 | 52.2 | | | 49.5 | | | | | 77.5 | | | | | | 31.3 | | | | | | | 72.8 | | | | | | 5 | | 56.6 | | | | | | 18.8 | | | | | 33.1% |
| 11 | 47.4 | | | 44.3 | | | | | 76.7 | | | | | | 29.8 | | | | | | | 60.7 | | | | | | 5 | | 51.8 | | | | | | 17.8 | | | | | 34.3% |
| 12 | 57.5 | | | 57.5 | | | | | 58.5 | | | | | | 27.4 | | | | | | | 63.2 | | | | | | 5 | | 52.8 | | | | | | 14.4 | | | | | 27.3% |
| 13 | 68.0 | | | 56.4 | | | | | 68.2 | | | | | | 30.6 | | | | | | | 75.4 | | | | | | 5 | | 59.7 | | | | | | 17.6 | | | | | 29.6% |
| 14 | 54.0 | | | 47.4 | | | | | 62.2 | | | | | | 23.9 | | | | | | | 62.6 | | | | | | 5 | | 50.0 | | | | | | 15.9 | | | | | 31.8% |
| 15 | 63.4 | | | 59.3 | | | | | 68.4 | | | | | | 27.1 | | | | | | | 68.0 | | | | | | 5 | | 57.3 | | | | | | 17.3 | | | | | 30.1% |
| 16 | 68.4 | | | 73.6 | | | | | 80.8 | | | | | | 33.6 | | | | | | | 79.9 | | | | | | 5 | | 67.3 | | | | | | 19.5 | | | | | 28.9% |
| **Cemdisiran (5 mg/kg) + Pozelimab (10 mg/kg)** | | | | | | | | | | | | | | | | | | | | | | | | | | | | | | | | | | | | | | | | | |
| **Time post-dose (weeks)** | **6001** | | | | | **6002** | | | | **6003** | | | | | **6004** | | | | | | | **6005** | | | | | | **n** | | **Mean** | | | | | | **STDEV** | | | | **%CV** | |
| 0 | 77.8 | | | | | 71.0 | | | | 65.6 | | | | | 66.8 | | | | | | | 74.4 | | | | | | 5 | | 71.1 | | | | | | 5.1 | | | | 7.2% | |
| 2 | 11.3 | | | | | 15.1 | | | | 9.7 | | | | | 21.2 | | | | | | | 7.8 | | | | | | 5 | | 13.0 | | | | | | 5.3 | | | | 40.7% | |
| 2.002976 | 12.2 | | | | | 16.1 | | | | 10.0 | | | | | 27.1 | | | | | | | 8.5 | | | | | | 5 | | 14.8 | | | | | | 7.4 | | | | 50.5% | |
| 2.02381 | 15.5 | | | | | 19.6 | | | | 12.9 | | | | | 30.5 | | | | | | | 8.9 | | | | | | 5 | | 17.5 | | | | | | 8.2 | | | | 47.2% | |
| 2.047619 | 13.4 | | | | | 18.4 | | | | 11.8 | | | | | 27.3 | | | | | | | 9.7 | | | | | | 5 | | 16.1 | | | | | | 7.0 | | | | 43.6% | |
| 2.142857 | 16.0 | | | | | 17.8 | | | | 11.1 | | | | | 25.8 | | | | | | | 10.8 | | | | | | 5 | | 16.3 | | | | | | 6.1 | | | | 37.7% | |
| 2.285714 | 20.7 | | | | | 23.7 | | | | 13.7 | | | | | 31.2 | | | | | | | 11.2 | | | | | | 5 | | 20.1 | | | | | | 8.0 | | | | 39.9% | |
| 2.428571 | 17.8 | | | | | 23.3 | | | | 12.1 | | | | | 28.0 | | | | | | | 9.0 | | | | | | 5 | | 18.0 | | | | | | 7.8 | | | | 43.4% | |
| 3 | 18.2 | | | | | 18.7 | | | | 9.8 | | | | | 24.9 | | | | | | | 9.3 | | | | | | 5 | | 16.2 | | | | | | 6.6 | | | | 40.7% | |
| 3.428571 | 19.9 | | | | | 18.2 | | | | 11.0 | | | | | 24.3 | | | | | | | 9.9 | | | | | | 5 | | 16.7 | | | | | | 6.1 | | | | 36.6% | |
| 4 | 21.6 | | | | | 17.2 | | | | 11.0 | | | | | 39.1 | | | | | | | 9.1 | | | | | | 5 | | 19.6 | | | | | | 12.0 | | | | 61.2% | |
| 4.571429 | 27.2 | | | | | 18.8 | | | | 10.9 | | | | | 39.8 | | | | | | | 11.0 | | | | | | 5 | | 21.5 | | | | | | 12.2 | | | | 56.7% | |
| 5 | 24.6 | | | | | 17.5 | | | | 11.5 | | | | | 34.2 | | | | | | | 11.6 | | | | | | 5 | | 19.9 | | | | | | 9.6 | | | | 48.4% | |
| 6 | 33.6 | | | | | 27.3 | | | | 13.7 | | | | | 28.2 | | | | | | | 16.9 | | | | | | 5 | | 23.9 | | | | | | 8.3 | | | | 34.8% | |
| 7 | 42.9 | | | | | 34.2 | | | | 17.0 | | | | | 36.0 | | | | | | | 30.3 | | | | | | 5 | | 32.1 | | | | | | 9.6 | | | | 29.8% | |
| 8 | 66.8 | | | | | 37.2 | | | | 20.9 | | | | | 32.9 | | | | | | | 25.6 | | | | | | 5 | | 36.7 | | | | | | 18.0 | | | | 49.0% | |
| 9 | 78.2 | | | | | 48.7 | | | | 28.0 | | | | | 44.8 | | | | | | | 43.6 | | | | | | 5 | | 48.7 | | | | | | 18.3 | | | | 37.6% | |
| 10 | 112.5 | | | | | 68.4 | | | | 47.1 | | | | | 53.7 | | | | | | | 51.3 | | | | | | 5 | | 66.6 | | | | | | 26.9 | | | | 40.4% | |
| 11 | 100.6 | | | | | 61.5 | | | | 44.5 | | | | | 58.0 | | | | | | | 68.8 | | | | | | 5 | | 66.7 | | | | | | 20.9 | | | | 31.4% | |
| 12 | 88.7 | | | | | 72.4 | | | | 42.3 | | | | | 52.6 | | | | | | | 91.4 | | | | | | 5 | | 69.5 | | | | | | 21.7 | | | | 31.2% | |
| 13 | 117.0 | | | | | 91.5 | | | | 74.9 | | | | | 60.6 | | | | | | | 69.0 | | | | | | 5 | | 82.6 | | | | | | 22.3 | | | | 27.0% | |
| 14 | 109.0 | | | | | 63.4 | | | | 53.5 | | | | | 55.5 | | | | | | | 48.1 | | | | | | 5 | | 65.9 | | | | | | 24.7 | | | | 37.5% | |
| 15 | 90.7 | | | | | 61.2 | | | | 52.5 | | | | | 67.4 | | | | | | | 55.0 | | | | | | 5 | | 65.4 | | | | | | 15.3 | | | | 23.4% | |
| 16 | 115.5 | | | | | 82.8 | | | | 68.0 | | | | | 76.8 | | | | | | | 67.9 | | | | | | 5 | | 82.2 | | | | | | 19.6 | | | | 23.9% | |
| **Cemdisiran (25 mg/kg) + Pozelimab (10 mg/kg)** | | | | | | | | | | | | | | | | | | | | | | | | | | | | | | | | | | | | | | | | | |
| **Time post-dose (weeks)** | **7001** | | **7002** | | | | | **7003** | | | | | | **7004** | | | | | | | **7005** | | | | | **n** | | | **Mean** | | | | | | **STDEV** | | | | **%CV** | | |
| 0 | 62.8 | | 65.7 | | | | | 58.1 | | | | | | 58.4 | | | | | | | 76.0 | | | | | 5 | | | 64.2 | | | | | | 7.3 | | | | 11.4% | | |
| 2 | 5.3 | | 3.6 | | | | | 5.5 | | | | | | 7.5 | | | | | | | 4.3 | | | | | 5 | | | 5.2 | | | | | | 1.5 | | | | 28.6% | | |
| 2.002976 | 5.9 | | 3.9 | | | | | 6.7 | | | | | | 11.9 | | | | | | | 5.6 | | | | | 5 | | | 6.8 | | | | | | 3.0 | | | | 44.5% | | |
| 2.02381 | 6.3 | | 4.5 | | | | | 7.0 | | | | | | 9.9 | | | | | | | 4.9 | | | | | 5 | | | 6.5 | | | | | | 2.2 | | | | 33.0% | | |
| 2.047619 | 6.2 | | 4.2 | | | | | 6.5 | | | | | | 9.4 | | | | | | | 4.4 | | | | | 5 | | | 6.2 | | | | | | 2.1 | | | | 33.8% | | |
| 2.142857 | 6.6 | | 4.2 | | | | | 8.2 | | | | | | 4.9 | | | | | | | 7.3 | | | | | 5 | | | 6.2 | | | | | | 1.7 | | | | 27.2% | | |
| 2.285714 | 7.0 | | 5.2 | | | | | 9.0 | | | | | | 9.6 | | | | | | | 4.9 | | | | | 5 | | | 7.1 | | | | | | 2.2 | | | | 30.2% | | |
| 2.428571 | 6.6 | | 3.7 | | | | | 8.9 | | | | | | 9.5 | | | | | | | 4.9 | | | | | 5 | | | 6.7 | | | | | | 2.5 | | | | 37.5% | | |
| 3 | 5.0 | | 3.8 | | | | | 8.3 | | | | | | 6.8 | | | | | | | 4.1 | | | | | 5 | | | 5.6 | | | | | | 1.9 | | | | 34.1% | | |
| 3.428571 | 5.6 | | 3.2 | | | | | 6.6 | | | | | | 6.0 | | | | | | | 4.3 | | | | | 5 | | | 5.1 | | | | | | 1.4 | | | | 26.9% | | |
| 4 | 4.9 | | 3.0 | | | | | 6.6 | | | | | | 6.3 | | | | | | | 3.6 | | | | | 5 | | | 4.9 | | | | | | 1.6 | | | | 32.0% | | |
| 4.571429 | 5.5 | | 3.7 | | | | | 6.6 | | | | | | 5.8 | | | | | | | 3.5 | | | | | 5 | | | 5.0 | | | | | | 1.4 | | | | 27.4% | | |
| 5 | 4.7 | | 3.7 | | | | | 6.2 | | | | | | 5.2 | | | | | | | 4.0 | | | | | 5 | | | 4.8 | | | | | | 1.0 | | | | 21.3% | | |
| 6 | 5.2 | | 4.3 | | | | | 6.9 | | | | | | 5.8 | | | | | | | 4.0 | | | | | 5 | | | 5.2 | | | | | | 1.2 | | | | 22.1% | | |
| 7 | 7.9 | | 6.1 | | | | | 7.7 | | | | | | 6.5 | | | | | | | 5.1 | | | | | 5 | | | 6.7 | | | | | | 1.2 | | | | 17.3% | | |
| 8 | 8.2 | | 8.2 | | | | | 10.2 | | | | | | 6.4 | | | | | | | 4.9 | | | | | 5 | | | 7.6 | | | | | | 2.0 | | | | 26.5% | | |
| 9 | 10.6 | | 9.2 | | | | | 10.9 | | | | | | 6.1 | | | | | | | 5.8 | | | | | 5 | | | 8.5 | | | | | | 2.4 | | | | 28.5% | | |
| 10 | 19.2 | | 16.4 | | | | | 14.4 | | | | | | 6.0 | | | | | | | 6.6 | | | | | 5 | | | 12.5 | | | | | | 6.0 | | | | 47.5% | | |
| 11 | 13.7 | | 17.5 | | | | | 16.4 | | | | | | 8.7 | | | | | | | 8.6 | | | | | 5 | | | 13.0 | | | | | | 4.2 | | | | 32.3% | | |
| 12 | 22.0 | | 20.6 | | | | | 15.3 | | | | | | 8.1 | | | | | | | 10.8 | | | | | 5 | | | 15.4 | | | | | | 6.0 | | | | 39.1% | | |
| 13 | 19.5 | | 29.0 | | | | | 21.0 | | | | | | 12.1 | | | | | | | 14.7 | | | | | 5 | | | 19.3 | | | | | | 6.5 | | | | 33.8% | | |
| 14 | 20.9 | | 28.0 | | | | | 26.3 | | | | | | 10.4 | | | | | | | 13.5 | | | | | 5 | | | 19.8 | | | | | | 7.7 | | | | 38.9% | | |
| 15 | 22.5 | | 39.5 | | | | | 25.5 | | | | | | 11.2 | | | | | | | 17.4 | | | | | 5 | | | 23.2 | | | | | | 10.6 | | | | 45.6% | | |
| 16 | 28.6 | | 50.5 | | | | | 29.3 | | | | | | 13.3 | | | | | | | 21.8 | | | | | 5 | | | 28.7 | | | | | | 13.8 | | | | 48.1% | | |

CV, coefficient of variation; STDEV, standard deviation.

Supplementary Table 4. Individual pozelimab/C5 molar ratios

| Pozelimab (5 mg/kg) | | | | | | | | | | | | | | | | | | | | | | | | | | | | | | | | | | | |  |  |
| --- | --- | --- | --- | --- | --- | --- | --- | --- | --- | --- | --- | --- | --- | --- | --- | --- | --- | --- | --- | --- | --- | --- | --- | --- | --- | --- | --- | --- | --- | --- | --- | --- | --- | --- | --- | --- | --- |
| Time post-dose (weeks) | | **3001** | | | | | **3002** | | | | **3003** | | | | **3004** | | | | **n** | | | **Mean** | | | | | **STDEV** | | | | | | **%CV** | | |  |  |
| 0 | |  | | | | |  | | | |  | | | |  | | | |  | | |  | | | | |  | | | | | |  | | |  |  |
| 0.002976 | | 0.03 | | | | | 0.02 | | | | 0.01 | | | | 0.01 | | | | 4 | | | 0.01 | | | | | 0.01 | | | | | | 57.02% | | |  |  |
| 0.02381 | | 0.18 | | | | | 0.15 | | | | 0.15 | | | | 0.14 | | | | 4 | | | 0.16 | | | | | 0.02 | | | | | | 10.70% | | |  |  |
| 0.047619 | | 0.30 | | | | | 0.28 | | | | 0.34 | | | | 0.28 | | | | 4 | | | 0.30 | | | | | 0.03 | | | | | | 9.94% | | |  |  |
| 0.142857 | | 0.44 | | | | | 0.45 | | | | 0.47 | | | | 0.47 | | | | 4 | | | 0.46 | | | | | 0.01 | | | | | | 3.26% | | |  |  |
| 0.285714 | | 0.45 | | | | | 0.43 | | | | 0.66 | | | | 0.48 | | | | 4 | | | 0.51 | | | | | 0.10 | | | | | | 20.65% | | |  |  |
| 0.428571 | | 0.56 | | | | | 0.55 | | | | 0.72 | | | | 0.53 | | | | 4 | | | 0.59 | | | | | 0.09 | | | | | | 14.54% | | |  |  |
| 1 | | 0.55 | | | | | 0.58 | | | | 0.60 | | | | 0.48 | | | | 4 | | | 0.55 | | | | | 0.05 | | | | | | 9.78% | | |  |  |
| 1.428571 | | 0.39 | | | | | 0.47 | | | | 0.48 | | | | 0.36 | | | | 4 | | | 0.43 | | | | | 0.06 | | | | | | 13.98% | | |  |  |
| 2 | | 0.36 | | | | | 0.39 | | | | 0.45 | | | | 0.31 | | | | 4 | | | 0.38 | | | | | 0.06 | | | | | | 14.65% | | |  |  |
| 2.571429 | | 0.30 | | | | | 0.35 | | | | 0.41 | | | | 0.27 | | | | 4 | | | 0.33 | | | | | 0.06 | | | | | | 18.48% | | |  |  |
| 3 | | 0.30 | | | | | 0.26 | | | | 0.41 | | | | 0.24 | | | | 4 | | | 0.30 | | | | | 0.08 | | | | | | 26.23% | | |  |  |
| 4 | | 0.23 | | | | | 0.21 | | | | 0.28 | | | | 0.17 | | | | 4 | | | 0.22 | | | | | 0.05 | | | | | | 21.78% | | |  |  |
| 5 | | 0.15 | | | | | 0.14 | | | | 0.22 | | | | 0.13 | | | | 4 | | | 0.16 | | | | | 0.04 | | | | | | 24.62% | | |  |  |
| 6 | | 0.09 | | | | | 0.08 | | | | 0.16 | | | | 0.07 | | | | 4 | | | 0.10 | | | | | 0.04 | | | | | | 37.62% | | |  |  |
| 7 | | 0.07 | | | | | 0.07 | | | | 0.11 | | | | 0.06 | | | | 4 | | | 0.08 | | | | | 0.02 | | | | | | 28.12% | | |  |  |
| 8 | | 0.05 | | | | | 0.05 | | | | 0.08 | | | | 0.04 | | | | 4 | | | 0.06 | | | | | 0.02 | | | | | | 29.67% | | |  |  |
| 9 | | 0.04 | | | | | 0.04 | | | | 0.04 | | | | 0.03 | | | | 4 | | | 0.04 | | | | | 0.01 | | | | | | 13.27% | | |  |  |
| 10 | | 0.02 | | | | | 0.02 | | | | 0.04 | | | | 0.02 | | | | 4 | | | 0.02 | | | | | 0.01 | | | | | | 46.15% | | |  |  |
| Pozelimab (10 mg/kg) | | | | | | | | | | | | | | | | | | | | | | | | | | | | | | | | | | | |  |  |
| Time post-dose (weeks) | **4001** | | | | | **4002** | | | | | **4003** | | | | **4004** | | | | **n** | | | | **Mean** | | | | | **STDEV** | | | | | **%CV** | | |  |  |
| 0 |  | | | | |  | | | | |  | | | |  | | | |  | | | |  | | | | |  | | | | |  | | |  |  |
| 0.002976 | 0.00 | | | | | 0.03 | | | | | 0.01 | | | | 0.02 | | | | 4 | | | | 0.01 | | | | | 0.01 | | | | | 68.49% | | |  |  |
| 0.02381 | 0.48 | | | | | 0.41 | | | | | 0.20 | | | | 0.31 | | | | 4 | | | | 0.35 | | | | | 0.12 | | | | | 34.17% | | |  |  |
| 0.047619 | 0.91 | | | | | 0.66 | | | | | 0.41 | | | | 0.71 | | | | 4 | | | | 0.67 | | | | | 0.21 | | | | | 30.85% | | |  |  |
| 0.142857 | 1.18 | | | | | 0.84 | | | | | 0.61 | | | | 0.99 | | | | 4 | | | | 0.91 | | | | | 0.24 | | | | | 26.37% | | |  |  |
| 0.285714 | 1.08 | | | | | 1.06 | | | | | 0.63 | | | | 0.93 | | | | 4 | | | | 0.93 | | | | | 0.21 | | | | | 22.59% | | |  |  |
| 0.428571 | 1.02 | | | | | 1.04 | | | | | 0.69 | | | | 1.02 | | | | 4 | | | | 0.94 | | | | | 0.17 | | | | | 17.96% | | |  |  |
| 1 | 1.04 | | | | | 1.06 | | | | | 0.65 | | | | 1.02 | | | | 4 | | | | 0.94 | | | | | 0.19 | | | | | 20.68% | | |  |  |
| 1.428571 | 0.90 | | | | | 0.80 | | | | | 0.56 | | | | 0.78 | | | | 4 | | | | 0.76 | | | | | 0.15 | | | | | 19.32% | | |  |  |
| 2 | 0.71 | | | | | 0.80 | | | | | 0.41 | | | | 0.66 | | | | 4 | | | | 0.64 | | | | | 0.17 | | | | | 26.18% | | |  |  |
| 2.571429 | 0.61 | | | | | 0.68 | | | | | 0.38 | | | | 0.59 | | | | 4 | | | | 0.57 | | | | | 0.13 | | | | | 22.69% | | |  |  |
| 3 | 0.54 | | | | | 0.55 | | | | | 0.29 | | | | 0.49 | | | | 4 | | | | 0.47 | | | | | 0.12 | | | | | 26.28% | | |  |  |
| 4 | 0.44 | | | | | 0.49 | | | | | 0.26 | | | | 0.36 | | | | 4 | | | | 0.39 | | | | | 0.10 | | | | | 25.98% | | |  |  |
| 5 | 0.29 | | | | | 0.36 | | | | | 0.22 | | | | 0.31 | | | | 4 | | | | 0.30 | | | | | 0.06 | | | | | 19.29% | | |  |  |
| 6 | 0.24 | | | | | 0.24 | | | | | 0.17 | | | | 0.24 | | | | 4 | | | | 0.22 | | | | | 0.03 | | | | | 15.13% | | |  |  |
| 7 | 0.14 | | | | | 0.16 | | | | | 0.09 | | | | 0.19 | | | | 4 | | | | 0.14 | | | | | 0.04 | | | | | 29.31% | | |  |  |
| 8 | 0.08 | | | | | 0.13 | | | | | 0.04 | | | | 0.12 | | | | 4 | | | | 0.09 | | | | | 0.04 | | | | | 45.29% | | |  |  |
| 9 | 0.09 | | | | | 0.11 | | | | | 0.02 | | | | 0.12 | | | | 4 | | | | 0.08 | | | | | 0.05 | | | | | 55.62% | | |  |  |
| 10 | 0.06 | | | | | 0.06 | | | | | 0.00 | | | | 0.07 | | | | 4 | | | | 0.05 | | | | | 0.03 | | | | | 61.87% | | |  |  |
| Cemdisiran (5 mg/kg) + Pozelimab (5 mg/kg) | | | | | | | | | | | | | | | | | | | | | | | | | | | | | | | | | | | | | |
| Time post-dose (weeks) | | | | **5001** | | | | | **5002** | | | | **5003** | | | | **5004** | | | | **5005** | | | | | **n** | | | **Mean** | | | **STDEV** | | | **%CV** | | |
| 0 | | | |  | | | | |  | | | |  | | | |  | | | |  | | | | |  | | |  | | |  | | |  | | |
| 2 | | | |  | | | | |  | | | |  | | | |  | | | |  | | | | |  | | |  | | |  | | |  | | |
| 2.002976 | | | | 0.02 | | | | | 0.02 | | | | 0.02 | | | | 0.10 | | | | 0.03 | | | | | 5 | | | 0.04 | | | 0.04 | | | 97.83% | | |
| 2.02381 | | | | 0.72 | | | | | 0.53 | | | | 0.61 | | | | 1.64 | | | | 1.15 | | | | | 5 | | | 0.93 | | | 0.46 | | | 49.84% | | |
| 2.047619 | | | | 1.42 | | | | | 1.35 | | | | 1.12 | | | | 3.44 | | | | 2.39 | | | | | 5 | | | 1.94 | | | 0.96 | | | 49.61% | | |
| 2.142857 | | | | 2.31 | | | | | 2.25 | | | | 1.35 | | | | 4.15 | | | | 3.33 | | | | | 5 | | | 2.68 | | | 1.08 | | | 40.37% | | |
| 2.285714 | | | | 2.89 | | | | | 2.92 | | | | 1.68 | | | | 3.97 | | | | 3.79 | | | | | 5 | | | 3.05 | | | 0.91 | | | 29.91% | | |
| 2.428571 | | | | 3.74 | | | | | 4.35 | | | | 2.09 | | | | 4.09 | | | | 3.96 | | | | | 5 | | | 3.65 | | | 0.90 | | | 24.58% | | |
| 3 | | | | 3.23 | | | | | 4.30 | | | | 2.22 | | | | 4.03 | | | | 4.08 | | | | | 5 | | | 3.57 | | | 0.86 | | | 23.97% | | |
| 3.428571 | | | | 3.03 | | | | | 3.34 | | | | 2.00 | | | | 3.73 | | | | 3.23 | | | | | 5 | | | 3.07 | | | 0.65 | | | 21.12% | | |
| 4 | | | | 2.69 | | | | | 2.61 | | | | 1.71 | | | | 2.92 | | | | 2.73 | | | | | 5 | | | 2.53 | | | 0.47 | | | 18.62% | | |
| 4.571429 | | | | 2.25 | | | | | 2.54 | | | | 1.33 | | | | 2.54 | | | | 2.03 | | | | | 5 | | | 2.14 | | | 0.50 | | | 23.33% | | |
| 5 | | | | 2.04 | | | | | 2.33 | | | | 1.37 | | | | 2.03 | | | | 2.13 | | | | | 5 | | | 1.98 | | | 0.36 | | | 18.28% | | |
| 6 | | | | 1.69 | | | | | 1.67 | | | | 1.03 | | | | 1.58 | | | | 1.28 | | | | | 5 | | | 1.45 | | | 0.29 | | | 19.79% | | |
| 7 | | | | 1.04 | | | | | 1.18 | | | | 0.75 | | | | 0.98 | | | | 0.92 | | | | | 5 | | | 0.97 | | | 0.16 | | | 16.29% | | |
| 8 | | | | 0.81 | | | | | 0.82 | | | | 0.54 | | | | 0.69 | | | | 0.63 | | | | | 5 | | | 0.70 | | | 0.12 | | | 16.84% | | |
| 9 | | | | 0.54 | | | | | 0.48 | | | | 0.36 | | | | 0.41 | | | | 0.45 | | | | | 5 | | | 0.45 | | | 0.07 | | | 15.53% | | |
| 10 | | | | 0.33 | | | | | 0.26 | | | | 0.19 | | | | 0.24 | | | | 0.21 | | | | | 5 | | | 0.25 | | | 0.05 | | | 21.34% | | |
| 11 | | | | 0.31 | | | | | 0.23 | | | | 0.14 | | | | 0.19 | | | | 0.19 | | | | | 5 | | | 0.21 | | | 0.06 | | | 29.98% | | |
| 12 | | | | 0.19 | | | | | 0.13 | | | | 0.13 | | | | 0.15 | | | | 0.14 | | | | | 5 | | | 0.15 | | | 0.03 | | | 17.73% | | |
| 13 | | | | 0.14 | | | | | 0.11 | | | | 0.09 | | | | 0.10 | | | | 0.08 | | | | | 5 | | | 0.11 | | | 0.02 | | | 22.84% | | |
| 14 | | | | 0.12 | | | | | 0.10 | | | | 0.08 | | | | 0.10 | | | | 0.07 | | | | | 5 | | | 0.09 | | | 0.02 | | | 21.06% | | |
| 15 | | | | 0.08 | | | | | 0.06 | | | | 0.05 | | | | 0.07 | | | | 0.05 | | | | | 5 | | | 0.06 | | | 0.01 | | | 17.54% | | |
| 16 | | | | 0.06 | | | | | 0.04 | | | | 0.03 | | | | 0.03 | | | | 0.03 | | | | | 5 | | | 0.04 | | | 0.01 | | | 28.54% | | |
| Cemdisiran (5 mg/kg) + Pozelimab (10 mg/kg) | | | | | | | | | | | | | | | | | | | | | | | | | | | | | | | | | | | | | |
| Time post-dose (weeks) | | | **6001** | | | | | **6002** | | | | **6003** | | | | **6004** | | | | **6005** | | | | | **n** | | | | **Mean** | | | **STDEV** | | | **%CV** | | |
| 0 | | |  | | | | |  | | | |  | | | |  | | | |  | | | | |  | | | |  | | |  | | |  | | |
| 2 | | |  | | | | |  | | | |  | | | |  | | | |  | | | | |  | | | |  | | |  | | |  | | |
| 2.002976 | | | 0.11 | | | | | 0.15 | | | | 0.17 | | | | 0.11 | | | | 0.13 | | | | | 5 | | | | 0.13 | | | 0.03 | | | 20.65% | | |
| 2.02381 | | | 1.72 | | | | | 3.07 | | | | 1.92 | | | | 0.82 | | | | 4.22 | | | | | 5 | | | | 2.35 | | | 1.32 | | | 56.03% | | |
| 2.047619 | | | 2.86 | | | | | 5.40 | | | | 3.57 | | | | 1.66 | | | | 7.29 | | | | | 5 | | | | 4.16 | | | 2.21 | | | 53.28% | | |
| 2.142857 | | | 4.43 | | | | | 7.58 | | | | 8.69 | | | | 3.72 | | | | 9.76 | | | | | 5 | | | | 6.83 | | | 2.65 | | | 38.73% | | |
| 2.285714 | | | 4.81 | | | | | 7.07 | | | | 10.29 | | | | 4.69 | | | | 12.16 | | | | | 5 | | | | 7.80 | | | 3.33 | | | 42.69% | | |
| 2.428571 | | | 6.91 | | | | | 7.12 | | | | 12.13 | | | | 5.41 | | | | 15.98 | | | | | 5 | | | | 9.51 | | | 4.41 | | | 46.41% | | |
| 3 | | | 7.31 | | | | | 7.62 | | | | 13.70 | | | | 5.41 | | | | 13.25 | | | | | 5 | | | | 9.46 | | | 3.77 | | | 39.82% | | |
| 3.428571 | | | 6.36 | | | | | 6.95 | | | | 11.09 | | | | 4.84 | | | | 10.80 | | | | | 5 | | | | 8.01 | | | 2.79 | | | 34.89% | | |
| 4 | | | 5.19 | | | | | 6.37 | | | | 10.44 | | | | 2.34 | | | | 10.00 | | | | | 5 | | | | 6.87 | | | 3.40 | | | 49.44% | | |
| 4.571429 | | | 3.55 | | | | | 5.29 | | | | 9.37 | | | | 1.73 | | | | 7.17 | | | | | 5 | | | | 5.42 | | | 2.99 | | | 55.15% | | |
| 5 | | | 3.48 | | | | | 5.00 | | | | 7.93 | | | | 1.83 | | | | 6.43 | | | | | 5 | | | | 4.93 | | | 2.40 | | | 48.59% | | |
| 6 | | | 2.03 | | | | | 2.35 | | | | 5.47 | | | | 1.82 | | | | 3.51 | | | | | 5 | | | | 3.04 | | | 1.51 | | | 49.70% | | |
| 7 | | | 1.39 | | | | | 1.59 | | | | 3.35 | | | | 1.19 | | | | 1.73 | | | | | 5 | | | | 1.85 | | | 0.86 | | | 46.70% | | |
| 8 | | | 0.78 | | | | | 1.12 | | | | 2.22 | | | | 0.93 | | | | 1.57 | | | | | 5 | | | | 1.32 | | | 0.58 | | | 44.07% | | |
| 9 | | | 0.53 | | | | | 0.71 | | | | 1.21 | | | | 0.47 | | | | 0.72 | | | | | 5 | | | | 0.73 | | | 0.29 | | | 39.88% | | |
| 10 | | | 0.30 | | | | | 0.38 | | | | 0.61 | | | | 0.33 | | | | 0.50 | | | | | 5 | | | | 0.42 | | | 0.13 | | | 30.05% | | |
| 11 | | | 0.26 | | | | | 0.33 | | | | 0.51 | | | | 0.24 | | | | 0.26 | | | | | 5 | | | | 0.32 | | | 0.11 | | | 35.65% | | |
| 12 | | | 0.22 | | | | | 0.20 | | | | 0.44 | | | | 0.20 | | | | 0.13 | | | | | 5 | | | | 0.24 | | | 0.12 | | | 50.54% | | |
| 13 | | | 0.13 | | | | | 0.13 | | | | 0.22 | | | | 0.12 | | | | 0.14 | | | | | 5 | | | | 0.15 | | | 0.04 | | | 29.13% | | |
| 14 | | | 0.10 | | | | | 0.16 | | | | 0.26 | | | | 0.10 | | | | 0.14 | | | | | 5 | | | | 0.15 | | | 0.06 | | | 42.21% | | |
| 15 | | | 0.08 | | | | | 0.13 | | | | 0.21 | | | | 0.06 | | | | 0.09 | | | | | 5 | | | | 0.11 | | | 0.06 | | | 50.51% | | |
| 16 | | | 0.05 | | | | | 0.07 | | | | 0.12 | | | | 0.03 | | | | 0.05 | | | | | 5 | | | | 0.06 | | | 0.03 | | | 51.66% | | |
| Cemdisiran (25 mg/kg) + Pozelimab (10 mg/kg) | | | | | | | | | | | | | | | | | | | | | | | | | | | | | | | | | | | | | |
| Time post-dose (weeks) | | | | | **7001** | | | | | **7002** | | | | **7003** | | | | **7004** | | | | | | **7005** | | | | | | **n** | **Mean** | | | **STDEV** | | | **%CV** |
| 0 | | | | |  | | | | |  | | | |  | | | |  | | | | | |  | | | | | |  |  | | |  | | |  |
| 2 | | | | |  | | | | |  | | | |  | | | |  | | | | | |  | | | | | |  |  | | |  | | |  |
| 2.002976 | | | | | 0.22 | | | | | 0.16 | | | | 0.19 | | | | 0.04 | | | | | | 0.27 | | | | | | 5 | 0.18 | | | 0.08 | | | 48.22% |
| 2.02381 | | | | | 5.54 | | | | | 5.33 | | | | 4.83 | | | | 2.31 | | | | | | 4.58 | | | | | | 5 | 4.52 | | | 1.30 | | | 28.67% |
| 2.047619 | | | | | 11.72 | | | | | 10.64 | | | | 10.70 | | | | 3.89 | | | | | | 9.63 | | | | | | 5 | 9.31 | | | 3.12 | | | 33.53% |
| 2.142857 | | | | | 15.97 | | | | | 18.91 | | | | 12.78 | | | | 15.61 | | | | | | 10.66 | | | | | | 5 | 14.79 | | | 3.17 | | | 21.43% |
| 2.285714 | | | | | 17.83 | | | | | 23.40 | | | | 16.82 | | | | 14.05 | | | | | | 24.63 | | | | | | 5 | 19.35 | | | 4.50 | | | 23.28% |
| 2.428571 | | | | | 19.13 | | | | | 35.71 | | | | 17.52 | | | | 13.84 | | | | | | 30.65 | | | | | | 5 | 23.37 | | | 9.33 | | | 39.92% |
| 3 | | | | | 23.93 | | | | | 33.33 | | | | 15.68 | | | | 18.42 | | | | | | 33.56 | | | | | | 5 | 24.98 | | | 8.28 | | | 33.12% |
| 3.428571 | | | | | 17.98 | | | | | 35.10 | | | | 17.34 | | | | 19.00 | | | | | | 27.72 | | | | | | 5 | 23.43 | | | 7.76 | | | 33.13% |
| 4 | | | | | 16.79 | | | | | 30.52 | | | | 16.32 | | | | 15.44 | | | | | | 29.15 | | | | | | 5 | 21.64 | | | 7.51 | | | 34.69% |
| 4.571429 | | | | | 13.23 | | | | | 23.59 | | | | 13.64 | | | | 13.82 | | | | | | 25.56 | | | | | | 5 | 17.97 | | | 6.08 | | | 33.82% |
| 5 | | | | | 14.51 | | | | | 21.10 | | | | 14.61 | | | | 13.87 | | | | | | 19.96 | | | | | | 5 | 16.81 | | | 3.43 | | | 20.40% |
| 6 | | | | | 9.18 | | | | | 15.06 | | | | 10.80 | | | | 10.56 | | | | | | 16.35 | | | | | | 5 | 12.39 | | | 3.12 | | | 25.21% |
| 7 | | | | | 4.85 | | | | | 9.45 | | | | 8.61 | | | | 8.35 | | | | | | 10.20 | | | | | | 5 | 8.29 | | | 2.06 | | | 24.80% |
| 8 | | | | | 3.24 | | | | | 5.57 | | | | 5.33 | | | | 5.84 | | | | | | 7.59 | | | | | | 5 | 5.51 | | | 1.55 | | | 28.16% |
| 9 | | | | | 1.84 | | | | | 4.11 | | | | 3.63 | | | | 4.41 | | | | | | 4.54 | | | | | | 5 | 3.71 | | | 1.10 | | | 29.73% |
| 10 | | | | | 0.72 | | | | | 1.92 | | | | 2.15 | | | | 3.57 | | | | | | 3.40 | | | | | | 5 | 2.35 | | | 1.17 | | | 49.76% |
| 11 | | | | | 0.90 | | | | | 1.54 | | | | 1.49 | | | | 1.91 | | | | | | 1.99 | | | | | | 5 | 1.57 | | | 0.43 | | | 27.64% |
| 12 | | | | | 0.34 | | | | | 0.99 | | | | 1.26 | | | | 1.60 | | | | | | 1.24 | | | | | | 5 | 1.09 | | | 0.47 | | | 43.27% |
| 13 | | | | | 0.32 | | | | | 0.52 | | | | 0.72 | | | | 0.81 | | | | | | 0.73 | | | | | | 5 | 0.62 | | | 0.20 | | | 31.67% |
| 14 | | | | | 0.29 | | | | | 0.48 | | | | 0.48 | | | | 0.74 | | | | | | 0.61 | | | | | | 5 | 0.52 | | | 0.17 | | | 32.20% |
| 15 | | | | | 0.21 | | | | | 0.32 | | | | 0.43 | | | | 0.57 | | | | | | 0.37 | | | | | | 5 | 0.38 | | | 0.13 | | | 35.28% |
| 16 | | | | | 0.12 | | | | | 0.22 | | | | 0.30 | | | | 0.42 | | | | | | 0.25 | | | | | | 5 | 0.26 | | | 0.11 | | | 42.14% |

CV, coefficient of variation; STDEV, standard deviation.
